# Beyond non-backtracking: non-cycling network centrality measures

**DOI:** 10.1098/rspa.2019.0653

**Published:** 2020-03-11

**Authors:** Francesca Arrigo, Desmond J. Higham, Vanni Noferini

**Affiliations:** 1 Department of Mathematics and Statistics, University of Strathclyde, Glasgow G1 1XH, UK; 2 School of Mathematics, University of Edinburgh, James Clerk Maxwell Building, Edinburgh EH9 3FD, UK; 3 Department of Mathematics and Systems Analysis, Aalto University, PO Box 11100, 00076 Aalto, Finland

**Keywords:** centrality index, deformed graph Laplacian, Hashimoto matrix, complex network, matrix polynomial, generating function

## Abstract

Walks around a graph are studied in a wide range of fields, from graph theory and stochastic analysis to theoretical computer science and physics. In many cases it is of interest to focus on non-backtracking walks; those that do not immediately revisit their previous location. In the network science context, imposing a non-backtracking constraint on traditional walk-based node centrality measures is known to offer tangible benefits. Here, we use the Hashimoto matrix construction to characterize, generalize and study such non-backtracking centrality measures. We then devise a recursive extension that systematically removes triangles, squares and, generally, all cycles up to a given length. By characterizing the spectral radius of appropriate matrix power series, we explore how the universality results on the limiting behaviour of classical walk-based centrality measures extend to these non-cycling cases. We also demonstrate that the new recursive construction gives rise to practical centrality measures that can be applied to large-scale networks.

## 1. Introduction

Our work is motivated by the wide range of areas in mathematics, computer science and physics where the concept of *non-backtracking* has proved useful, including spectral graph theory [1–3], number theory [4], discrete mathematics [5,6], quantum chaos [7], random matrix theory [8], stochastic analysis [9], applied linear algebra [10] and computer science [11,12]. In particular, non-backtracking has recently been introduced in the field of network science, where it has been shown to form the basis of effective algorithms for finding communities [13,14], optimizing percolation, [15,16], comparing networks [17,18] and assigning centrality values to nodes [13,14,19–25].

A key novelty in our work is to *extend the concept of non-backtracking to the case of non-triangulating, non-squaring and generally the avoidance of all cycles*. To make the idea practical, we develop an appropriate recursive extension to the Hashimoto matrix construction which allows the required quantities to be computed via matrix powering and projection. We study theoretical properties of the resulting network centrality measures and show that they can be applied to large-scale datasets. Because the basic Hashimoto matrix construction is not standard in graph theory, and has been derived from different viewpoints in other fields, we give in §1a a simple motivating illustration. This allows us to explain the notation and set up the main combinatoric task.

### (a) Illustration

**Figure 1.**
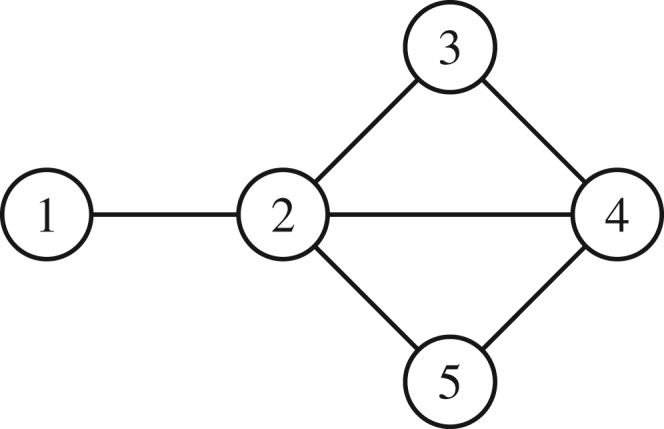
An undirected graph with five nodes.

Figure 1 shows an undirected, unweighted graph with five nodes. It is convenient to regard each undirected edge as a reciprocal pair of directed edges. We write *i* → *j* to denote the directed edge from node *i* to node *j*, so, for example, the connection between nodes 1 and 2 in figure 1 gives rise to 1 → 2 and 2 → 1. The adjacency matrix for this graph has the form

$$A=\left[\begin{array}{ccccc} 0 & 1 & 0 & 0 & 0\\ 1 & 0 & 1 & 1 & 1\\ 0 & 1 & 0 & 1 & 0\\ 0 & 1 & 1 & 0 & 1\\ 0 & 1 & 0 & 1 & 0 \end{array}\right]\in R^{5\times 5}.$$

Here, for example, *a*_1,2_ = 1 because there is an edge 1 → 2 and *a*_1,3_ = 0 because there is no edge 1 → 3.

A *walk* around a graph is any route from node to node that makes use of the available edges. The adjacency matrix provides a convenient way to count walks. For example the fourth power of *A* has its 1, 5 entry equal to 6 because there are six distinct walks of length four (that is, using four edges) starting at node 1 and finishing at node 5: these are 1 → 2 → 1 → 2 → 5, 1 → 2 → 3 → 2 → 5, 1 → 2 → 3 → 4 → 5, 1 → 2 → 4 → 2 → 5, 1 → 2 → 5 → 2 → 5 and 1 → 2 → 5 → 4 → 5. Generally (*A*^*r*^)_*ij*_ counts the number of distinct walks of length *r* starting at node *i* and finishing at node *j*; see, for example, [26, theorem 2.2.1].

An operation which is central to our work is the construction of the *line graph* [27]. Here, edges in the original graph are regarded as nodes in the corresponding line graph. Nodes *i* → *j* and *k* → *l* in this new line graph are connected if *j* = *k*, that is, if, together, they represent a walk of length two in the original graph.

For illustration, we show in table 1 the entries in the adjacency matrix for the line graph of the graph in figure 1. Here we have chosen a specific ordering of the edges in the original graph. Zero entries have been left blank. We will denote this 12 × 12 matrix by *W*. Note that *W* is not symmetric; for example, *w*_1→2, 2→3_ = 1 but *w*_2→3, 1→2_ = 0. Essentially, *W* is encoding the presence of walks of length two in the original graph. Its second power, *W*^2^, then counts walks of length three. For example, by definition,

$${\left(W^{2}\right)}_{1\to 2,\;3\to 4}=\sum_{a\to b}w_{1\to 2,\;a\to b}\;w_{a\to b,\;3\to 4},$$

which reduces to 1 because the only nonzero product in the sum arises from *w*_1→2, 2→3_ *w*_2→3, 3→4_, corresponding to the walk 1 → 2 → 3 → 4 in the original graph. Similarly, *W*^3^ counts walks of length four in the original graph. For example,

$${\left(W^{3}\right)}_{1\to 2,\;4\to 2}=\sum_{a\to b}\sum_{c\to d}w_{1\to 2,\;a\to b}\;w_{a\to b,\;c\to d}\;w_{c\to d,\;4\to 2},$$

which equals 2 because of the existence of the two walks 1 → 2 → 3 → 4 → 2 and 1 → 2 → 5 → 4 → 2 in the original graph.

**Table 1.** Adjacency matrix for the line graph of the graph in figure 1. Entries that represent backtracking are starred.

	1 → 2	2 → 1	2 → 3	3 → 2	2 → 4	4 → 2	2 → 5	5 → 2	3 → 4	4 → 3	4 → 5	5 → 4
1 → 2		$1^{⋆}$	1		1		1					
2 → 1	$1^{⋆}$											
2 → 3				$1^{⋆}$					1			
3 → 2		1	$1^{⋆}$		1		1					
2 → 4						$1^{⋆}$				1	1	
4 → 2		1	1		$1^{⋆}$		1					
2 → 5								$1^{⋆}$				1
5 → 2		1	1		1		$1^{⋆}$					
3 → 4						1				$1^{⋆}$		
4 → 3				1					$1^{⋆}$			
4 → 5								1				$1^{⋆}$
5 → 4						1				1	$1^{⋆}$	

Generally, the *r*th power of *W* counts walks of length *r* + 1 in the original graph. Because of our choice of labelling, the second, third, fifth and seventh rows of *W*^*r*^ record walks starting with an edge of the form 2→⋆, and the first, fourth, sixth and eighth columns record walks that end with an edge of the form ⋆→2. It follows that by taking linear combinations of the appropriate rows and columns we can recover the node-based counts for walks starting at node 2 and finishing at node 2. Similar remarks apply for all nodes, and hence an appropriate linear projection of *W*^*r*^ recovers all the walk count information in *A*^*r*+1^. This fact is formalized in part (i) of proposition 2.4.

From the perspective of this work, a major benefit of the line graph setting is that we may modify the adjacency matrix *W* in a way that allows us to count only non-backtracking walks; that is, walks which never leave a node and then immediately return to it. In table 1 the starred entries represent reciprocated pairs of edges, such as 1 → 2 and 2 → 1. Replacing all such entries by zero, thereby creating the *Hashimoto matrix* or *non-backtracking matrix* [28] and calling this new matrix *B*, it follows that powers of *B* will automatically count non-backtracking walks, and the same projection method gives node-based results; see part (ii) of proposition 2.4.

The remainder of the manuscript is organized as follows. In §2 we set up the full notation, discuss relevant network centrality measures and describe the benefits that have been found to arise when non-backtracking is introduced. Section 3 then exploits the Hashimoto matrix approach in order to characterize non-backtracking centrality measures based on general Taylor Series expansions. For such measures, it is of interest to characterize universality behaviour arising at the radius of convergence, and in §4 we study this issue. In §5 we then develop and analyse a recursive strategy that promotes non-backtracking into non-triangulating, non-squaring, and, generally, the removal of all cycles. Having derived the new construction, we consider computational complexity issues and then analyse the universality behaviour. Section 6 gives the results of computational experiments that illustrate the feasibility of non-cycling centrality measures on real networks.

## 2. Preliminary material

Our fundamental object of study is an undirected graph. However, as illustrated in §1a, the operations that we apply will typically generate new directed graphs. Hence, we give definitions for the general case of a directed graph G=(V,E), with unweighted edges, and no self-loops or multiple edges. We denote by *n* the number of nodes and by *m* the number of edges.

**Remark 2.1.**

For undirected graphs, we interpret each undirected edge *i* − *j* as a pair of directed edges *i* → *j* and *j* → *i*, and we denote by *m* the total number of such directed edges.

The graph G can be represented by means of its *adjacency matrix* $A=(a_{ij})\in R^{n\times n}$, whose nonzero entries are *a*_*ij*_ = 1 if and only if i→j∈E. The matrix *A* thus contains *m* nonzeros, one for each edge in the graph. We use *I*, **1** and **0** to denote the identity matrix, the vector of ones and the vector of zeros, respectively, and a subscript will indicate the dimension where this is not obvious. For every edge i→j∈E we will call *i* the *source node* of the edge and *j* the *target node* of the edge. The edge *j* → *i* will be referred to as the *reciprocal* of edge *i* → *j*. The number $d_{i}^{\text{out}}$ of edges originating from node *i* will be referred to as the *out-degree* of node *i*, while the number $d_{i}^{\text{in}}$ of edges targeting node *i* will be referred to as the *in-degree* of node *i*. For undirected graphs, $d_{i}^{\text{out}}=d_{i}^{\text{in}}=:d_{i}$ for all nodes i∈V and this common value is usually referred to as the *degree* of node *i*. A bold font denotes a vector, so *d*_*i*_ is the *i*th component of **d**. A *walk* of length *r* is a sequence of *r* + 1 nodes *i*_1_, *i*_2_, …, *i*_*r*+1_ such that $i_{\ell }\to i_{\ell +1}\in E$ for all ℓ = 1, …, *r*. A walk is said to be *backtracking* if it uses a consecutive pair of reciprocal edges and *non-backtracking* otherwise. We will use the acronyms NBT and NBTW for non-backtracking and non-backtracking walk. A *path* is a walk with no repeated nodes, with the only possible exception of the first and last nodes. If these coincide the path is then called a *cycle*. We mentioned in §1a that the entries of the *r*th power of *A* record the number of walks of length *r*; that is, (*A*^*r*^)_*ij*_ is the number of distinct walks of length *r* from node *i* to node *j*, for all *r* = 0, 1, …. Following [24], we denote by *p*_*r*_(*A*) the non-backtracking analogue of *A*^*r*^, so the (*i*, *j*)th entry of *p*_*r*_(*A*) contains the number of NBTWs of length *r* from node *i* to node *j*. We use the convention that *p*_0_(*A*) = *I*. It is readily seen that *p*_1_(*A*) = *A* and *p*_2_(*A*) = *A*^2^ − *D*, where *D* is the diagonal matrix such that *D*_*ii*_ = (*A*^2^)_*ii*_. It has been proved [5,10] that for all *r* ≥ 0 the matrices *p*_*r*+3_(*A*) satisfy a four-term recurrence when *A*^*T*^ ≠ *A*:

$$p_{r+3}(A)=p_{r+2}(A)A+p_{r+1}(A)(I-D)-p_{r}(A)(A-A\circ A^{T}),$$

where ° denotes the Schur (entrywise) product.

In the undirected setting, where *A* = *A*^*T*^, this reduces to a three-term recurrence [6]: for all *r* ≥ 1,

2.1 $$p_{r+2}(A)=p_{r+1}(A)A+p_{r}(A)(I-D).$$

As in the example of §1a, we denote by $W\in R^{m\times m}$ the adjacency matrix whose entries are *W*_*i*→*j*,ℓ→*h*_ = *δ*_*j*ℓ_, where *δ*_*j*ℓ_ is the Kronecker delta. So *W* represents a network with *m* nodes, each corresponding to an edge in G, and a connection exists between two nodes if the corresponding edges in G are such that the target node of the first coincides with the source node of the second, and the two edges thus form a walk of length two in G. We refer to *W* as the *edge-matrix*. If G is undirected, then *W* is the adjacency matrix of the *line graph* corresponding to G. Finally, we denote by $B\in R^{m\times m}$ the non-backtracking version of *W*; that is, the adjacency matrix of the network obtained by connecting two of the *m* nodes, each corresponding to an edge in G, if and only if the corresponding two edges form a NBTW of length two in G. We note that *B* = *W* − *W*° *W*^*T*^. We may aso write

2.2 $$B_{i\to j,\ell \to h}=\delta_{j\ell }(1-\delta_{ih}).$$

The matrix *B* is often referred to as the *Hashimoto matrix* [28] or the *non-backtracking edge-matrix*.

### (a) Equivalent centrality vectors

A central issue in network science is to determine the most important players within the graph. This activity has applications in a wide range of areas, ranging from social science, marketing and politics to epidemiology and well-being [27,29]. The problem may be tackled using *centrality measures*. These functions, which are invariant under relabelling of the nodes in the graph, assign to each node a non-negative number that quantifies its importance—the higher the value, the more important the node. We take the standard viewpoint that the value assigned to each node is not interesting *per se*; we are concerned with the ranking that arises. It thus follows that two centrality vectors that assign different values to the nodes but induce the same ranking are *equivalent*. In this sense, it is worth pointing out that neither shifting a centrality measure with a uniform vector nor multiplying a centrality measure with a positive scalar changes the ranking of the nodes. We may, indeed, define an equivalence class among centrality vectors as follows. Let $\text{u},\text{v}\in R^{n}$ be two non-negative, non-zero vectors. Then

2.3 $$\text{u}∼\text{v}\;\Leftrightarrow \;\exists \;\alpha >0,\beta \geq (-\min_{i}{\left(\text{v}\right)}_{i})\;\text{such that}:\;\text{u}=\alpha (\text{v}+\beta 1).$$

Two different representatives of the same equivalence class yield the same node ranking. We note in passing that one could consider a smaller collection of equivalence classes, e.g.two measures are equivalent if they induce the same ranking. However, for the purposes of this work, restricting our study to (2.3) suffices to compare the rankings induced by parametric matrix functions and those induced by their limits.

Many centrality measures have been introduced over the years. In this work we focus on the very broad class of walk based centrality measures induced by functions [30–32]

2.4 $$f(z)=\sum_{r=0}^{\infty }c_{r}z^{r}\in P,$$

where P is the set of functions analytic in a neighbourhood of zero that can be expressed with a Maclaurin series with non-negative coefficients *c*_*r*_ for all *r* = 0, 1, …

Clearly, *f*(*z*) = *e*^*z*^ and *f*(*z*) = (1 − *z*)^−1^ belong to P. We will denote by *ρ*_*f*_ the radius of convergence of the series *f*(*z*), which can be finite or infinite.

For a function f∈P defined on the spectrum of a matrix *A*, we will refer to (*f*(*tA*))_*ii*_ as the *f*-subgraph centrality of node *i*, and to (*f*(*tA*)**1**)_*i*_ as the *f*-total communicability of node *i*. Here, *t* > 0 is a parameter that we are free to choose, with the constraint that the power series must converge. From the power series expansion of *f* and from the fact that powers of *A* count walks of given lengths, it follows that the *f*-subgraph centrality of a node measures how strongly each node is involved in closed walks of any length; similarly, its *f*-total node communicability measures how well this node communicates with all the nodes in the network. We note that in the classic Katz case, where *f*(*z*) = (1 − *z*)^−1^, the parameter *t* represents an attenuation factor that downweights walks of length *k* by a factor *t*^*k*^ [33], and hence, in a message-passing setting, *t* may be viewed as the probability of successfully traversing an edge.

These concepts can be extended to the framework of NBTWs, by defining the *NBT *f*-subgraph centrality* and *NBT *f*-total (node) communicability* of node *i* as

2.5 $$x{\left(t\right)}_{i}={\left(\sum_{r=0}^{\infty }c_{r}\;t^{r}\;p_{r}(A)\right)}_{ii}\;\text{and}\;y{\left(t\right)}_{i}={\left(\sum_{r=0}^{\infty }c_{r}\;t^{r}\;p_{r}(A)1\right)}_{i},$$

respectively, for non-negative coefficients *c*_*r*_.

It is intuitively reasonable that eliminating backtracking walks, and hence focusing on traversals that explore the network more widely, should lead to an improved centrality measure in applications where a message-passing or disease-spreading analogy is relevant. In the case of *random walks*, it is known that NBTWs mix faster [9]. For NBT generalizations of Katz centrality, three concrete benefits have been identified:

— *Localization:* Suppose we have a family of non-negative unit Euclidean norm vectors $x\in R^{n}$, defined for all large *n*. Then, from [34], the *inverse participation ratio* is defined to be $S(x):=\sum_{i=1}^{n}x_{i}^{4}$. The family of vectors is said to be *localized* if S=O(1) and *nonlocalized* if S=o(1), as *n* → ∞. Intuitively, localization implies that the majority of the mass in the vector is confined to a finite subset of components. When **x** is a centrality measure, localization corresponds to the undesirable circumstance where the algorithm has focused almost exclusively on a subset of the network, and does not give useful information about the relative importance of the majority of nodes. In this context, the effect was first highlighted in [25]. Numerical tests in [19,24] showed that a NBT version of the standard Katz algorithm [33] avoids localization effects observed for Katz on a range of real networks. Furthermore, rigorous asymptotic analysis on specific network classes backed up these results; for example, in [19] for a directed windmill network with an arbitrary number of blades.
— *Range of parameter values:* The classic Katz centrality measure [27,29,33] assigns the value ((*I* − *t A*)^−1^**1**)_*i*_ to node *i*. To produce a well-defined non-negative measure, the downweighting parameter *t* must lie in the range 0 < *t* < 1/*ρ*(*A*), where *ρ*( · ) denotes the spectral radius. For the NBT version *y*_*i*_(*t*) in (2.5) with *c*_*r*_ ≡ 1, it was shown in [24] that *t* must be chosen in the range 0 < *t* < 1/*ρ*(*C*), where
  2.6 $$C=\left[\begin{array}{ccc} A & \left(I-D\right) & \left(A\circ A^{T}-A\right)\\ I & 0 & 0\\ 0 & I & 0 \end{array}\right]\in R^{3n\times 3n}.$$
  By construction, since the NBT count cannot exceed the standard walk count, this radius of convergence must be larger than the upper limit 1/*ρ*(*A*) for Katz. In practice, the difference can be significant, and hence NBT Katz can support a much greater choice of downweighting parameters, allowing global features of the network to have a stronger influence on the measure.
*Pruning:* From a practical viewpoint, it appears that *ρ*(*C*) in (2.6) must be computed, or approximated, in order to determine an appropriate range of *t* values. However, it was shown in [19] that *ρ*(*C*) does not change when *leaves*, *source nodes* and *dangling nodes* are removed. (The equivalent statement is not true for standard Katz, where *ρ*(*A*) is not invariant to such deletions.) Moreover, these operations can be performed recursively until no such nodes exist. On realistic networks, these low cost pruning steps were found to reduce the typical network size by around 30%, making NBT Katz more efficient than standard Katz.

It is also of interest to characterize the form of these centrality measures at their radius of convergence; for example, it was also shown in [19] that the NBT eigenvector approach proposed in [25] arises as the limiting case *t* → 1/*ρ*(*C*)^−^ in NBT Katz.

Our aim here is to show how NBTW-based measures can be studied, and generalized, by working in the edge space and then projecting. We will show that this approach allows us to (1) unify and extend the current theory, yielding results that hold for any analytic function, (2) describe limiting behaviour at the radius of convergence, and (3) extend to the case where walks avoid triangles, squares and, generally, all cycles up to any fixed length.

### (b) Source and target matrices

We now collect together some results that allow us to perform projections from the edge space onto the node space. Many of these results can be found in disparate areas of the literature, especially for the case of undirected graphs, [35,36]. We present and justify them here because they form the core of our analysis.

**Definition 2.2.**

Let G be an unweighted, possibly directed, graph with *n* nodes and *m* edges. Its *source* and *target* (or *terminal*) matrices $L,R\in R^{m\times n}$ are entrywise defined as:

$$L_{ei}=\left\{ \begin{array}{ll} 1 & \text{if edge}\;e\;\text{has the form}\;i\to ⋆\\ 0 & \text{otherwise} \end{array}\right.\;\text{and}\;R_{ej}=\left\{ \begin{array}{ll} 1 & \text{if edge}\;e\;\text{has the form}\;⋆\to j\\ 0 & \text{otherwise} \end{array}\right.,$$

respectively, for all *e* = 1, 2, …, *m* and for all *i*, *j* = 1, 2, …, *n*.

Note that both *L* and *R* have precisely one nonzero element equal to 1 in every row. This identifies the source/target node of the corresponding edge; hence, *L***1**_*n*_ = *R***1**_*n*_ = **1**_*m*_. We also note in passing that, for directed graphs, the matrix *L* − *R* is an incidence matrix.

Proposition 2.3 recalls some basic properties of the source and target matrices [35,36].

**Proposition 2.3.**

*Let* G *be an unweighted, directed, graph with no self-loops nor multiple edges. Then, in the above notation, L*^*T*^ *R* = *A*, *R L*^*T*^ = *W*, *L*^*T*^ *L is diagonal with the out degrees on the diagonal, and R*^*T*^ *R is diagonal with the in degrees on the diagonal. If the network is undirected, then R*^*T*^*R* = *L*^*T*^*L* = *D*.

**Proof.**

The results can be proved entrywise from the definition of the source and target matrices.

For example, to confirm the first equality we note that for all i,j∈V it holds that ${\left(L^{T}R\right)}_{ij}=\sum_{e=1}^{m}L_{ei}R_{ej}=1$ if and only if there is an edge from node *i* to node *j* and is 0 otherwise. So (*L*^*T*^ *R*)_*ij*_ = *a*_*ij*_. ▪

The following proposition summarizes useful properties of the source and target matrices and, in particular, shows that they can be used to move from the edge space to the node space.

**Proposition 2.4.**

*Let* G *be an unweighted, possibly directed, graph with no self-loops nor multiple edges. Then*

(i) *L*^*T*^ *W*^*r*^ *R* = *A*^*r*+1^, *for all* *r* = 0, 1, …;
(ii) *L*^*T*^ *B*^*r*^ *R* = *p*_*r*+1_(*A*), *for all* *r* = 0, 1, …;
(iii) *L*^*T*^ (*W*^*T*^° *W*) *R* = *R*^*T*^(*W*^*T*^° *W*)*L* = *D, where D is diagonal with D*_*ii*_ = (*A*^2^)_*ii*_;
(iv) *R*^*T*^ *W L is a diagonal matrix whose i*^*th*^ *diagonal element is* $d_{i}^{\text{in}}d_{i}^{\text{out}}$ ($=\;d_{i}^{2},$ *if* G *is undirected*);
(v) *R*^*T*^ *B L is a diagonal matrix whose i*^*th*^ *diagonal element is equal to the number of NBTWs of length two of the form* ⋆→i→⋆.

**Proof.**

(i) We proceed by induction. The result has been proved for *r* = 0 in proposition 2.3. Suppose that *L*^*T*^ *W*^*r*−1^ *R* = *A*^*r*^ up to a certain *r* ≥ 1, then from proposition 2.3 *L*^*T*^ *W*^*r*^ *R* = *L*^*T*^ *W*^*r*−1^ *R L*^*T*^ *R* = *A*^*r*^ *A* = *A*^*r*+1^.
(ii) Let us first note that $B_{ef}^{r}$ is the number of NBTWs of length *r* + 1 in G starting with edge *e* and ending with edge *f*. Then ${\left(L^{T}B^{r}R\right)}_{ij}=\sum_{e=1}^{m}\sum_{f=1}^{m}L_{ei}B_{ef}^{r}R_{fj}$ is the number of NBTWs of length *r* + 1 starting from node *i* and ending at node *j*, which is *p*_*r*+1_(*A*).
(iii) Exploiting the fact that *p*_2_(*A*) = *A*^2^ − *D* it follows that *L*^*T*^ (*W*^*T*^° *W*) *R* = *L*^*T*^ (*W* − *B*) *R* = *A*^2^ − *p*_2_(*A*) = *D*. Moreover, *R*^*T*^ (*W*° *W*^*T*^) *L* = (*L*^*T*^ (*W*^*T*^° *W*) *R*)^*T*^ = *D*.
(iv) The result follows directly from proposition 2.3.
(v) For all i,j∈V we have that ${\left(R^{T}BL\right)}_{ij}=\sum_{e=1}^{m}\sum_{f=1}^{m}R_{ei}B_{ef}L_{fj}$ counts the number of NBTWs of length two formed by edges *e* and *f*, and such that *e* targets node *i* and *f* originates from node *j*. Clearly, this sum is always zero, unless *i* = *j*, and in this case the sum equals the number of NBTWs of length two through node *i*. ▪

In the next section, we describe how to exploit the matrices *L* and *R* to compute the NBTW generating function induced by any analytic function. We emphasize that our basic object of study in the remainder of this work is an undirected network, so that *A* = *A*^*T*^ and the matrices *p*_*r*_(*A*) satisfy the recurrence (2.1), but directed networks will arise when we use the Hashimoto construction and its extensions.

## 3. Projection techniques for non-backtracking centralities

Consider a function f(z)∈P in (2.4), which we recall is analytic in a neighbourhood of zero, with *c*_*r*_ > 0 for all *r* and radius of convergence *ρ*_*f*_. Define the linear operator ∂ acting on *f* as follows:

$$\partial f(z):=\sum_{r=0}^{\infty }c_{r+1}z^{r}=\left\{ \begin{array}{ll} \frac{f(z)-f(0)}{z} & \text{ if }z\neq 0;\\ f^{\prime }(0) & \text{ if }z=0, \end{array}\right.$$

so that ∂f(z)∈P.

Before stating our first results on projection techniques for computing non-backtracking walk based centrality measures, let us remark that, since *A* is symmetric, its spectrum is real. Moreover, the spectrum of *W* will be real also, even though *W* is not symmetric in general. Indeed, from proposition 2.3 we know that *A* = *LR*^*T*^ and *W* = *R*^*T*^*L*, and Flanders Theorem [37, theorem 2] implies that the spectrum of *W* coincides with that of *A*, up to the multiplicity of 0.

Let us also recall that the spectrum of *B* coincides with the reversal (e.g. [38]) of that of the symmetric matrix polynomial [39] *M*(*t*) = *I* − *tA* + *t*^2^(*D* − *I*), i.e. that of the deformed graph Laplacian; e.g. [6,24] and references therein. We note that the reversal of the deformed graph Laplacian has been called Bethe-Hessian by some authors [40,41]. In [24] it was also shown that for every *λ* in the spectrum of *M*(*t*), we have |*λ*| ≥ 1/*ρ*(*A*). (Below, in proposition 5.9, we will improve this result and show that the inequality is always strict for a non-empty graph.)

In the remainder of this paper, we will often implicitly make use of the following classical result; see, for example, [42, theorem 4.7].

**Theorem 3.1.**

*Suppose f has a Taylor series expansion*

$$f(z)=\sum_{r=0}^{\infty }c_{r}{\left(z-z_{0}\right)}^{r}\;\left(c_{r}=\frac{f^{r}(z_{0})}{r!}\right)$$

*with radius of convergence ρ*_*f*_. *If* $A\in C^{n\times n}$, *then f*(*A*) *is well defined and is given by*

$$f(A)=\sum_{r=0}^{\infty }c_{r}{\left(A-z_{0}I\right)}^{r}$$

*if and only if each of the distinct eigenvalues* *λ*_1_, …, *λ*_*s*_ *of A satisfies one of the conditions*:

(i) |*λ*_*i*_ − *z*_0_| < *ρ*_*f*_;
(ii) |*λ*_*i*_ − *z*_0_| = *ρ*_*f*_ *and the series for* $f^{\left(n_{i}-1\right)}(\lambda )$, *where n*_*i*_ *is the index of λ*_*i*_, *is convergent at λ* = *λ*_*i*_ *for i* = 1, …, *s*.

Finally, let us state here the following simple consequence of the Cauchy–Hadamard theorem [43, theorem 3.39], which relates the radii of convergence of *f* and ∂*f*.

**Lemma 3.2.**

*Let* $f(z)=\sum_{r=0}^{\infty }c_{r}z^{r}$ and let $\partial f(z)=\sum_{r=0}^{\infty }c_{r+1}z^{r}.$ *Then, f*(*z*) *converges for* |*z*| < *ρ*_*f*_ *if and only if* ∂*f*(*z*) *converges for* |*z*| < *ρ*_*f*_.

Using these remarks and the results from the previous section, we may prove the following.

**Theorem 3.3.**

*Let* G *be an unweighted, possibly directed, graph with no self-loops nor multiple edges. In the above notation, for* 0 < *t* < *ρ*_*f*_/*ρ*(*A*) *it holds that*

$$\sum_{r=0}^{\infty }c_{r}t^{r}A^{r}=c_{0}I+tL^{T}[\partial f(tW)]R$$

*and*

3.1 $$\sum_{r=0}^{\infty }c_{r}t^{r}p_{r}(A)=c_{0}I+tL^{T}[\partial f(tB)]R.$$

**Proof.**

From the definition of ∂*f* it follows that

$$\partial f(tW)=\sum_{r=0}^{\infty }c_{r+1}t^{r}W^{r}\;\text{and}\;\partial f(tB)=\sum_{r=0}^{\infty }c_{r+1}t^{r}B^{r},$$

implying by (*i*) and (*ii*) in proposition 2.4 that

$$tL^{T}[\partial f(tW)]R=\sum_{r=0}^{\infty }c_{r+1}t^{r+1}A^{r+1}\;\text{and}\;tL^{T}[\partial f(tB)]R=\sum_{r=0}^{\infty }c_{r+1}t^{r+1}p_{r+1}(A),$$

and thus the conclusion. ▪

Theorem 3.3 has several implications. For example, in the framework of undirected networks, setting *f*(*z*) = (1 − *z*)^−1^ and observing that ∂*f*(*z*) = *f*(*z*), we obtain for 0 < *t* < 1/*ρ*(*A*)

3.2*a* $$I_{n}+tL^{T}[{\left(I-tW\right)}^{-1}]R={\left(I-tA\right)}^{-1}$$

and

3.2*b* $$I_{n}+tL^{T}[{\left(I-tB\right)}^{-1}]R=\sum_{r=0}^{\infty }t^{r}p_{r}(A)=(1-t^{2})M{\left(t\right)}^{-1},$$

where *M*(*t*) = *I* − *tA* − *t*^2^(*I* − *D*) is the deformed graph Laplacian of the network. We note that the second equality in (3.2b) was proved in [24]. These results give an equivalence in the sense of (2.3) between Katz centrality on *W* projected through *L*^*T*^ and Katz centrality on *A* (3.2a), and between NBT resolvent based centrality on *A* and Katz centrality on *B* projected via *L*^*T*^ (3.2b); indeed, since *R***1**_*n*_ = **1**_*m*_, we have

3.3*a* $$1_{n}+tL^{T}{\left(I-tW\right)}^{-1}1_{m}={\left(I-tA\right)}^{-1}1_{n}$$

and

3.3*b* $$1_{n}+tL^{T}{\left(I-tB\right)}^{-1}1_{m}=(1-t^{2})M{\left(t\right)}^{-1}1_{n}.$$

More generally, theorem 3.3 implies that we can compute the NBTW generating function associated with (2.4) via (3.1), and thus rewrite (2.5), for appropriate values of *t*, as

$$x{\left(t\right)}_{i}=c_{0}+t{\left(L{\text{e}}_{i}\right)}^{T}\partial f(tB)(R{\text{e}}_{i})\;\text{and}\;y{\left(t\right)}_{i}=c_{0}+t{\left(L{\text{e}}_{i}\right)}^{T}\partial f(tB)1_{m}.$$

This approach induces a duality operation on graphs as described in table 2, which, however, is not invertible; indeed, the dual of the dual graph is not the primal graph.

**Table 2.** Relationships between walks and matrices in the primal and dual spaces.

primal	dual
edge, i.e. ones in *A*	node
walk of length 2	edge, i.e. ones in *W*
NBTW of length 2	nonreciprocal edge, i.e. ones in *B*
BTW of length 2	reciprocal edge, i.e. ones in *W* − *B* = *W*^*T*^° *W*
*A*^*r*+1^	*W*^*r*^
*p*_*r*+1_(*A*)	*B*^*r*^

It was shown in [19,20,24] that there are more direct ways to compute NBTW centrality measures that do not rely on this projection technique. However, as we show below, this approach has the advantages of (1) being simpler to describe for a general *f*(*z*), (2) unifying the theory, so that universality results may be studied, and (3) extending to walks that do not allow for cycles up to any fixed length.

## 4. Limiting behaviour and universality

It is well known that the classic Katz centrality measure becomes equivalent in the sense of (2.3) to the so-called eigenvector centrality measure [44] as the downweighting parameter approaches its upper limit, and becomes equivalent to degree centrality as the downweighting parameter approaches zero [27]. In [45] the authors derived a general set of such results for walk-based centrality measures. Here, we show how to obtain non-backtracking versions of these results via the Hashimoto matrix construction.

We begin by relating the (left and right) Perron eigenvectors of *B* and the NBT eigenvector; that is, the eigenvector of *M*(*t*) associated with the smallest eigenvalue. (Note that *M*(*t*) is symmetric, and hence its left and right eigenvectors are the same.) Throughout this work, $t\to t^{⋆}$ for any $t^{⋆}>0$ is taken to be the limit from below, and *t* → 0 is taken to be the limit from above.

**Theorem 4.1.**

*Let A be the adjacency matrix of a simple, connected network with at least two cycles. Let B be its Hashimoto matrix and M*(*t*) *be its deformed graph Laplacian. Moreover, let* $\omega \in R^{n}$ *and* $\text{z},\text{w}\in R^{m}$ *be non-negative vectors with* $||\text{w}|{|}_{1}=||\text{z}|{|}_{1}=||\omega |{|}_{1}=1$ *such that M*(*μ*)**ω** = **0**, *μB***w** = **w**, and *μ***z**^*T*^*B* = **z**^*T*^, *where μ is the smallest eigenvalue of M*(*t*). *Then*,

$$\omega =L^{T}\text{w}=R^{T}\text{z}.$$

**Proof.**

Observe first that by [24, proposition 7.5] *μ* is a simple eigenvalue of *M*(*t*) and *I* − *tB* (both seen as matrix polynomials), and that it is their smallest. Let us decompose both *M*(*t*) = *M*(*t*)^*T*^ and *I* − *tB* via the respective analytic SVDs:

$$M(t)=U_{M}(t)\Sigma_{M}(t)U_{M}{\left(t\right)}^{T},\;\;I-tB=U_{B}(t)\Sigma_{B}(t)V_{B}{\left(t\right)}^{T}.$$

Denote now by, respectively, **u**_*M*,*n*_(*t*), **v**_*B*,*m*_(*t*), **u**_*B*,*m*_(*t*), *σ*_*M*,*n*_(*t*), *σ*_*B*,*m*_(*t*) the last columns of *U*_*M*_(*t*), *V*_*B*_(*t*), *U*_*B*_(*t*) and the last diagonal elements of Σ_*M*_(*t*) and Σ_*B*_(*t*). Then, arguing similarly to the proofs of [19, theorem 6.1] and [24, theorem 10.1] we have the expansions

$$\begin{array}{ll} & (1-t^{2})M{\left(t\right)}^{-1}1_{n}=\frac{(1-t^{2})u_{M,n}{\left(t\right)}^{T}1_{n}}{\sigma_{M,n}(t)}u_{M,n}(t)+O({\left(t-\mu \right)}^{0}),\\ & {\left(I-tB\right)}^{-1}1_{m}=\frac{u_{B,m}{\left(t\right)}^{T}1_{m}}{\sigma_{B,m}(t)}v_{B,m}(t)+O({\left(t-\mu \right)}^{0})\\ \text{and} & {\left(I-tB^{T}\right)}^{-1}1_{m}=\frac{v_{B,m}{\left(t\right)}^{T}1_{m}}{\sigma_{B,m}(t)}u_{B,m}(t)+O({\left(t-\mu \right)}^{0}). \end{array}$$

Moreover, it holds that

$$\lim_{t\to \mu }{\text{u}}_{M,n}(t)=\frac{\omega }{||\omega |{|}_{2}},\;\lim_{t\to \mu }{\text{u}}_{B,m}(t)=\frac{\text{z}}{||\text{z}|{|}_{2}},\;\lim_{t\to \mu }{\text{v}}_{B,m}(t)=\frac{\text{w}}{||\text{w}|{|}_{2}}.$$

Multiplying (3.3b) by *σ*_*M*,*n*_(*t*) and taking the limit *t* → *μ* we see that there exists α∈R, *α* ≠ 0, such that **ω** = *αL*^*T*^**w**. Moreover, since *L*, **w**, **ω** are all non-negative, we have *α* > 0. Similarly, multiplying the transpose of (3.3b) by *σ*_*M*,*n*_(*t*) and taking the limit *t* → *μ* we see that there exists *β* > 0 such that **ω** = *βR*^*T*^**z**. To conclude the proof, note that since **w**, **z** ≥ **0** and $||\text{w}|{|}_{1}=||\text{z}|{|}_{1}=1$, the fact that *L*, *R* have precisely one element equal to 1 in each row yields $||L^{T}\text{w}|{|}_{1}=||R^{T}\text{z}|{|}_{1}=1$, and thus *α* = *β* = 1. ▪

We note that we are correcting here a typo in the proof of theorem 6.1 in [19].

We now consider the case where there is a single cycle present within the graph.

**Lemma 4.2.**

*Let B be the Hashimoto matrix of a simple, connected graph that contains precisely one cycle. Then*:

(i) *The Perron eigenvalue of B is* 1 *and it has geometric and algebraic multiplicity two. Moreover, suppose that we label the edges within four sets as follows: first all the edges going through the cycle in one direction, which, without loss of generality, we call counterclockwise; then all the edges going through the cycle clockwise; then all the edges not on the cycle (if any) going towards the cycle; finally all the edges not on the cycle (if any) going away from the cycle. Then, partitioning according to these four sets, a basis for* ker⁡(B−I) *is given by*
  $$F=\left[\begin{array}{cc} 1 & 0\\ 0 & 1\\ 1 & 1\\ 0 & 0 \end{array}\right]\in R^{m\times 2}.$$
(ii) *We have*
  $$L^{T}F=\left[\begin{array}{cc} 1 & 1 \end{array}\right]\in R^{n\times 2}.$$

**Proof.**

(i) That 1 is the Perron eigenvalue of *B*, and that its algebraic multiplicity is two, is a consequence of [2, equation (2.3) and corollary 1] and [24, lemma 6.2]. Note that (*BF*)_*e*1_ is equal to the number of NBTWs of length two over edge *e* and either an edge that goes through the cycle counterclockwise or an edge that goes towards the cycle. This number is 1 if edge *e* either goes counterclockwise through the cycle or goes towards the cycle, and it is 0 otherwise. Similarly (*BF*)_*e*2_ counts NBTWs of length two that consist of edge *e* and an edge that either goes clockwise through the cycle or goes towards the cycle. This is 1 if edge *e* either goes clockwise through the cycle or points towards the cycle, and 0 otherwise. We conclude that *BF* = *F*. Moreover, manifestly *F* has rank two. Hence the geometric multiplicity of the eigenvalue 1 is exactly two, as this cannot exceed the algebraic multiplicity.
(ii) By definition of *L* and *F*, the (*i*, 1)th element of *L*^*T*^ *F* counts how many edges, among those either in the cycle and going counterclockwise or not on the cycle and going towards it, start from node *i*. There is precisely one such edge for all *i*. Replacing ‘counterclockwise’ with ‘clockwise’, the same argument shows that (*L*^*T*^ *F*)_*i*2_ = 1. ▪

We now prove a universality result for NBTW-based centralities that generalizes the Katz version in [24, theorem 10.1] and echoes the result presented in [45] for classical centralities. Recall that the equivalence relation is defined in (2.3).

**Theorem 4.3.**

*Let* $f(z)=\sum_{r}c_{r}z^{r}$ *with c*_*r*_ > 0 *for all r and with radius of convergence ρ*_*f*_, *and suppose that f*(*ρ*_*f*_) *diverges. Let A be the adjacency matrix of a simple and connected graph, B its Hashimoto matrix and* $t\in (0,\bar{t}),$ *with* $\bar{t}=\rho_{f}/\rho (B).$ *Then the NBT f-subgraph centrality vector* **x**(*t*) *in* (*2.5*) *and the NBT* *f-total communicability vector* **y**(*t*) *in* (*2.5*) *are such that*

$$\text{x}(t\to 0)∼\left\{ \begin{array}{ll} 1 & \text{if the graph is a tree}\\ {\text{d}}^{\left(\ell \right)} & \text{otherwise} \end{array}\right.\;\text{and}\;\text{y}(t\to 0)∼\text{d},$$

*where* ℓ > 2 *is the length of the shortest cycle in the graph (if any)*, **d**^(ℓ)^ *is the vector whose ith entry is the number of cycles of length ℓ involving node i, and* **d** *is the vector of degrees. Moreover*,

(i) *if the graph contains at least two cycles, then*
  $$\text{x}(t\to \bar{t})∼\omega \circ \omega \;\text{and}\;\text{y}(t\to \bar{t})∼\omega ,$$
  *where **ω** is as in theorem* 4.1.
(ii) *if the graph contains exactly one cycle, then* $x{\left(t\to \bar{t}\right)}_{i}$ *depends only on the distance of node i from the cycle and*
  $$\text{y}(t\to \bar{t})∼1;$$
(iii) *if the graph is a tree, then* $\bar{t}=\infty$ *and*
  $$\text{x}(t\to \bar{t})∼1\;\text{and}\;\text{y}(t\to \bar{t})∼p_{\kappa }(A)1,$$
  *where κ is the length of the longest non-backtracking walk in the graph*.

**Proof.**

We may obtain *t* → 0 limits directly from the series expansions. We begin by considering **x**(*t*). If the graph is a tree, there are no closed walks and thus **x**(*t* → 0) = *c*_0_**1** ∼ **1**. Suppose now that there is at least one cycle in the graph and that the length of the shortest cycle is ℓ > 2. Then working entrywise, for all *i* = 1, 2, …, *n* we have

$${x(t)}_{i}=c_{0}+c_{1}tA_{ii}+c_{2}t^{2}{\left(A^{2}-D\right)}_{ii}+c_{3}t^{3}p_{3}{\left(A\right)}_{ii}+\cdots =c_{0}+c_{\ell }t^{\ell }p_{\ell }{\left(A\right)}_{ii}+\sum_{r>\ell }c_{r}t^{r}p_{r}{\left(A\right)}_{ii}.$$

Letting **p**^(*r*)^ be the vector whose *i*th entry is the element *p*_*r*_(*A*)_*ii*_ for all *r* ≥ 3, we have

$$\text{x}(t)∼\frac{\text{x}(t)-c_{0}1}{c_{\ell }t^{\ell }}={\text{p}}^{\left(\ell \right)}+\sum_{r>\ell }\frac{c_{r}}{c_{\ell }}t^{r-\ell }{\text{p}}^{\left(r\right)}\to {\text{p}}^{\left(\ell \right)}.$$

Finally, we note that since there are no cycles of length <ℓ, then **p**^(ℓ)^ = **d**^(ℓ)^. The result **y**(*t* → 0) ∼ **d** follows similarly.

We now prove the statements about the upper limit.

(i) If the graph contains at least two cycles, then **ρ*(*B*) > 1* [24]. From theorem 3.3 it follows that
  4.1a $$x{\left(t\right)}_{i}∼{\text{e}}_{i}^{T}L^{T}\left(\sum_{r=0}^{\infty }c_{r+1}t^{r}B^{r}\right)R{\text{e}}_{i}={\text{e}}_{i}^{T}L^{T}\partial f(tB)R{\text{e}}_{i}$$
  and similarly, using the fact that *R***1**_*n*_ = **1**_*m*_,
  4.1b $$\text{y}(t)∼L^{T}\partial f(tB)1_{m}.$$
  By lemma 3.2 and standard results in matrix theory, the matrix function ∂*f*(*t B*) has the same radius of convergence of *f*(*t B*), that is, $\bar{t}=\rho_{f}/\rho (B)$. To study the limit $t\to \bar{t}$, we can use the definition of a matrix function based on the Jordan decomposition of a matrix [42, Definition 1.2]. This leads to an argument along the lines of [45, theorem 5.2]. (Note that *B* can be taken, with no loss of generality, to be irreducible, see [24, proof of proposition 7.5]; note also the subtleties discussed in [45, remark 1] necessary to deal with the case when *B* is imprimitive.) Since this is a standard approach, we skip the details and describe the result: given the left and right Perron eigenvectors of the Hashimoto matrix **w**, $\text{z}\in R^{m}$, normalized so that $||\text{w}|{|}_{1}=||\text{z}|{|}_{1}=1$, we have
  $$\partial f(tB)=\gamma \partial f(\rho_{f}t/\bar{t})\text{w}{\text{z}}^{T}+O(1)$$
  for some *γ* > 0. Using the latter equality, equation (4.1a), and theorem 4.1 it follows that for all *i* = 1, 2, …, *n*
  $$x{\left(t\to \bar{t}\right)}_{i}∼({\text{e}}_{i}^{T}L^{T}\text{w})({\text{z}}^{T}R{\text{e}}_{i})=\omega_{i}^{2},$$
  where *M*(1/*ρ*(*B*))**ω** = **0**, and thus $\text{x}(t\to \bar{t})∼\omega \circ \omega$. Similarly, since $||\text{z}|{|}_{1}=1$ and **z** ≥ **0**, we have
  $$\text{y}(t\to \bar{t})∼L^{T}\text{w}=\omega .$$
(ii) If the graph contains precisely one cycle, then **ρ*(*B*) = 1* has geometric multiplicity 2 and, up to relabelling of the nodes, *F* defined as in lemma 4.2 is a basis for ker⁡(B−I). Hence, for some $Z\in R^{m\times 2}$, when *t* → 1 we have
  $$\partial f(tB)=\partial f(t)F\Gamma Z^{T}+O(1),$$
  where *Z* ≥ 0 and $\Gamma \in R^{2\times 2}$. Using $L^{T}F=[1_{n}\;1_{n}]$ from lemma 4.2, we see from (4.1b) that
  $$\text{y}(t\to 1)∼[1_{n}\;1_{n}]\Gamma Z^{T}1_{n},$$
  so **y**(*t* → 1) ∼ **1**_*n*_. For the *f*-subgraph centrality, note that
  $$x{\left(t\to 1\right)}_{i}∼\sum_{r=3}^{\infty }c_{r}{\left(p_{r}(A)\right)}_{ii}.$$
  Suppose that the unique cycle has length ℓ and that node *i* has a distance of *k* edges from the cycle (*k* = 0 if node *i* belongs to the cycle): then, for *r* ≥ 1,
  $${\left(p_{r}(A)\right)}_{ii}=\left\{ \begin{array}{ll} 2 & \mathrm{if}\;r=2k+h\ell \;\mathrm{for}\;\mathrm{some}\;h\geq 1\\ 0 & \mathrm{otherwise}. \end{array}\right.$$
  Hence,
  $$x{\left(t\to 1\right)}_{i}∼\sum_{r=3}^{\infty }c_{r}{\left(p_{r}(A)\right)}_{ii}=2\sum_{h=1}^{\infty }c_{2k+h\ell }.$$
(iii) If the graph is a tree, i.e. it does not contain any cycle, then *ρ*(*B*) = 0 and thus $\bar{t}=\infty$. Moreover, *p*_*r*_(*A*)_*ii*_ = 0 for all *r* ≥ 1 and for all *i*, so that **x**(*t*) = *c*_0_**1** and hence **x**(*t* → ∞) ∼ **1**. Since the graph is a tree, it also follows that the matrix power series is a polynomial in *t*; let *κ* be the length of the longest non-backtracking walk in the graph, i.e. the diameter of the graph. Then *p*_*r*_(*A*) = 0 for all *r* > *κ* and thus
  $$\text{y}(t\to \infty )∼p_{\kappa }(A)1_{n}.$$

 ▪

Theorem 4.3 highlights very different behaviour of the two types of centrality. It is intuitively clear that the NBT constraint in *f*-total communicability should become irrelevant as *t* → 0; here walks of length one dominate, and these never backtrack. However, for *f*-subgraph centrality, the shortest closed walks under the NBT constraint are cycles of length ℓ > 2. Theorem 4.3 shows that in the generic case where the graph contains at least two cycles, as $t\to \bar{t}$ both centrality measures converge to an equivalent of the projection of the Perron eigenvector of *M*(*t*) obtained via *L*^*T*^. In the specific cases when the graph either contains exactly one cycle or none, we again have a mismatch between the limiting behaviour of the two NBT *f*-centrality measures. The qualitatively different behaviour when there are two or more cycles is intuitively explained by the fact that the presence of at least two cycles allows us to “change direction” when walking around the network. If the graph contains only one cycle, then in the edge-space we have two connected components, one corresponding to the cycle being visited clockwise and one corresponding to the cycle being visited counterclockwise. On the other hand, if we have two cycles, then in the edge-space we have one strongly connected component instead of two.

## 5. Beyond non-backtracking: non-*k*-cycling

The projection approach described in §2b is based on a duality relation on graphs that builds on the source and target matrices associated with the adjacency matrix *A*. We now show how this approach can be iterated to compute weighted sums of walks that do not backtrack and do not contain any cycle of length up to a given *k*.

We therefore define the matrices $p_{r;k}(A)\in R^{n\times n}$, whose (*i*, *j*) elements count walks of length *r* from node *i* to node *j* which do not backtrack and do not allow for cycles of length up to *k*. Our aim is to study generalizations of (2.5) to the case of the non-backtracking and up to non-*k*-cycling *f*-subgraph centrality measure **x**_*k*_(*t*) and *f*-total communicability measure **y**_*k*_(*t*), defined entry-wise for *i* = 1, …, *n* as

5.1 $${\left({\text{x}}_{k}(t)\right)}_{i}={\left(\sum_{r=0}^{\infty }c_{r}t^{r}p_{r;k}(A)\right)}_{ii}\;\text{and}\;{\left({\text{y}}_{k}(t)\right)}_{i}={\left(\sum_{r=0}^{\infty }c_{r}t^{r}p_{r;k}(A)1\right)}_{i}.$$

**Remark 5.1.**

Because a closed walk of length *r* must contain a cycle of length no more than *r*, it follows that the sum defining (**x**_*k*_(*t*))_*i*_ in (5.1) may be taken from *r* = *k* + 1; the terms from *r* = 0 to *r* = *k* are zero. So, although the subgraph centrality concept is based on counting closed walks, the non-cycling constraint rules out all such walks that are deemed to be too short.

Throughout this section, we will adopt the notation (*i*_1_, *i*_2_, …, *i*_*r*_) to denote the walk *i*_1_ → *i*_2_ → · · · → *i*_*r*_ of length *r* − 1 in the original graph and we will denote by i the corresponding multi-index. We remark that open walks of length ℓ that do not backtrack and do not include any cycle are *open paths* of length ℓ.

The following matrix allows us to perform the iterative computations.

**Definition 5.2 (Non-*k*-cycling matrix).**

For *k* = 1, the non-*k*-cycling matrix, *P*_*k*_, corresponds to the adjacency matrix *A*. For *k* = 2 the matrix *P*_*k*_ corresponds to the Hashimoto matrix *B* in (2.2). More generally, for *k* > 2 the matrix *P*_*k*_ has as many rows and columns as the number of open paths of length *k* − 1 in the original graph. The entry ${\left(P_{k}\right)}_{ij}$ takes the value 1 if the original graph admits an open path starting at node *i*_1_ and finishing at node *j*_*k*_ which superposes with i on its last *k* − 1 steps and superposes with j on its first *k* − 1 steps. Hence, for any two open paths $i=(i_{1},\ldots ,i_{k})$ and $j=(j_{1},\ldots ,j_{k})$ of length *k* − 1 ≥ 1 we have

$${\left(P_{k}\right)}_{ij}=\left\{ \begin{array}{ll} 1 & \text{if}\;i_{r}=j_{r-1}\;\text{for}\;r=2,\ldots ,k\;\text{and}\;i_{1}\neq j_{k}\\ 0 & \text{otherwise.} \end{array}\right.$$

The next result shows how *P*_*k*_ may be constructed. We emphasize that *k* = 2 corresponds to the NBTW setting; see also [35,36]

**Theorem 5.3.**

*Let W*_1_ = *A be the adjacency matrix of a simple graph. Then, for k* ≥ 2 *the non-k-cycling matrix P*_*k*_ *can be recursively computed as*

$$P_{k}=W_{k}-\Delta_{k},$$

*where* $W_{k}=R_{k-1}L_{k-1}^{T},$ $\Delta_{k}=W_{k}\circ {\left(W_{k}^{T}\right)}^{k-1}$ *and L*_*k*−1_ *and R*_*k*−1_ *are the source and target matrix of the graph whose adjacency matrix is P*_*k*−1_.

**Remark 5.4.**

We note that the matrices *W*_*k*_ in the statement of theorem 5.3 correspond to the adjacency matrices of the *k*th order De Bruijn graphs of paths in the network; see [46].

**Proof.**

We first argue that the dimension of *W*_*k*_ matches that of *P*_*k*_. By construction *W*_2_ corresponds to the edge-matrix, *W*, which contains as many rows and columns as the number of edges in the (directed) graph represented by *W*_1_ = *A*. For *k* > 2, the matrix *W*_*k*_ has as many rows and columns as the number of open paths of length *k* − 1 in the original graph. Moreover, each row in the matrix *L*_*k*−1_ (resp., *R*_*k*−1_) corresponds to a walk of length *k* − 1, say (*i*_1_, …, *i*_*k*_), that neither backtracks nor contains cycles of length up to *k* − 1. Furthermore, such a row will contain a 1 in the entry corresponding to the column associated with the non-backtracking and up to non-(*k* − 2)-cycling path (*i*_1_, …, *i*_*k*−1_) (resp, (*i*_2_, …, *i*_*k*_)). The entries of $W_{k}=R_{k-1}L_{k-1}^{T}$ will then equal one if and only if the two paths of length *k* − 1 corresponding to the row and column indices under consideration are such that the last *k* − 2 edges of the first path coincide with the first *k* − 2 of the second path, and thus they form a path of length *k*. In summary: for any two paths $i=(i_{1},\ldots ,i_{k})$ and $j=(j_{1},\ldots ,j_{k})$ of length *k* − 1 ≥ 1 it holds that

$${\left(W_{k}\right)}_{ij}=\left\{ \begin{array}{ll} 1 & \text{if}\;i_{r}=j_{r-1}\;\text{for}\;r=2,\ldots ,k\\ 0 & \text{otherwise.} \end{array}\right.$$

By construction the matrix *W*_*k*_ will contain a one where two paths of length (*k* − 1) form a cycle of length *k*. It is therefore clear that the matrix *P*_*k*_ will be obtained from *W*_*k*_ by removing such entries. Hence, to complete the proof we must show that $\Delta_{k}=W_{k}\circ {\left(W_{k}^{T}\right)}^{k-1}$ identifies cycles of length *k* in the original graph. To do so, note that

$${\left({\left(W_{k}^{T}\right)}^{k-1}\right)}_{ij}=\sum_{h^{\left(1\right)},h^{\left(2\right)},\ldots ,h^{\left(k-2\right)}}{\left(W_{k}\right)}_{jh^{\left(1\right)}}{\left(W_{k}\right)}_{h^{\left(1\right)}h^{\left(2\right)}}\cdots {\left(W_{k}\right)}_{h^{\left(k-2\right)}i}.$$

Considering each term in the product on the right-hand side individually, we see from the definition of *W*_*k*_ that the first term will equal 1 if and only if $j_{r}=h_{r-1}^{\left(1\right)}$ for *r* = 2, …, *k*. The second term will equal 1 if and only if $h_{r}^{\left(1\right)}=h_{r-1}^{\left(2\right)}$ for *r* = 2, …, *k*, and this also implies the product of the first two terms will equal 1 if and only if $j_{r}=h_{r-2}^{\left(2\right)}$ for *r* = 3, …, *k*. Proceeding in this way, the (*k* − 1)th term will be non zero if and only if $h_{r}^{\left(k-3\right)}=h_{r-1}^{\left(k-2\right)}$ for *r* = 2, …, *k*, so that the product of the first (*k* − 1) terms will equal 1 when $j_{r}=h_{r-k+2}^{\left(k-2\right)}$ for *r* = *k* − 1, *k*. Finally, the last condition is for the last term to equal one, and this happens when $h_{r}^{\left(k-2\right)}=i_{r-1}$ for *r* = 2, …, *k*. Therefore, the product of all terms will equal one if and only if *j*_*r*_ = *i*_*r*−*k*+1_ for *r* = *k*, i.e. when *j*_*k*_ = *i*_1_. Moreover note that, for two given paths i and j there will be at most one non-zero product in the summation, since we only need to check that the final node in the first walk coincides with the first node in the second walk. Therefore,

$${\left({\left(W_{k}^{T}\right)}^{k-1}\right)}_{ij}=\left\{ \begin{array}{ll} 1 & \text{if}\;j_{k}=i_{1}\\ 0 & \text{otherwise.} \end{array}\right.$$

Exploiting the definition of *W*_*k*_ and ${\left(W_{k}^{T}\right)}^{k-1}$ it follows that Δ_*k*_ will have a 1 in position (i,j) if and only if

$$i_{1}=j_{k}⟶i_{2}=j_{1}⟶\ldots ⟶i_{k}=j_{k-1}⟶i_{1}=j_{k},$$

i.e. if the two paths form a (directed) cycle of length *k* in the original graph. This concludes the proof. ▪

It follows from the definition of *P*_*k*_ that taking a step in its associated graph corresponds to taking a NBT and up to non-*k*-cycling walk of length *k* in the original network; more generally, taking *r* consecutive steps within the graph associated to *P*_*k*_ corresponds to taking *k* + *r* − 1 steps in the original graph, while avoiding backtracking and cycles of up to length *k*.

Using the left and right projectors

5.2 $$L_{k-1}=L_{k-1}\cdots L_{1},\;\;R_{k-1}=R_{k-1}\cdots R_{1},$$

it is immediately clear that the following theorem holds.

**Theorem 5.5.**

*For all r* = 0, 1, … *and for any given k* ≥ 2, *we have*

$$L_{k-1}^{T}(P_{k}^{r})R_{k-1}=p_{r+k-1;k}(A).$$

**Remark 5.6.**

When *k* = 2, then *p*_*r*;*k*_(*A*) = *p*_*r*_(*A*) and theorem 5.5 reduces to proposition 2.4 (ii).

In order to obtain useful expressions for **x**_*k*_(*t*) and **y**_*k*_(*t*) in (5.1), we first study the generating function

5.3 $$\Phi_{k}(t)=\sum_{r=0}^{\infty }c_{r}t^{r}p_{r;k}(A).$$

Note that, given a certain *k*, for walks of length *r* ≤ *k* − 1 it holds that *p*_*r*;*k*_(*A*) = *p*_*r*;*r*_(*A*), since no cycles of length *k* can be formed using less than *k* edges. Therefore, our problem reduces to that of computing

5.4 $${\hat{\Phi }}_{k}(t)=\sum_{r=k-1}^{\infty }c_{r}t^{r}p_{r;k}(A),$$

which implicitly yields all the *p*_*r*;*k*_(*A*) for the interesting case *r* ≥ *k*.

Our procedure for computing ${\hat{\Phi }}_{k}(t)$ is the following. From theorem 5.5 it follows that

$$L_{k-1}^{T}[\partial^{k-1}f(tP_{k})]R_{k-1}=\sum_{r=0}^{\infty }c_{r+k-1}t^{r}p_{r+k-1;k}(A)=\sum_{r=k-1}^{\infty }c_{r}t^{r-k+1}p_{r;k}(A)$$

and thus

$$t^{k-1}L_{k-1}^{T}[\partial^{k-1}f(tP_{k})]R_{k-1}={\hat{\Phi }}_{k}(t).$$

Hence, having obtained *P*_*k*_ from the construction in theorem 5.3, we may obtain ${\hat{\Phi }}_{k}(t)$ as follows: (i) Compute ∂^*k*−1^*f*(*tP*_*k*_); (ii) Project: $L_{k-1}^{T}[\partial^{k-1}f(tP_{k})]R_{k-1}$; (iii) Multiply by *t*^*k*−1^.

Here, in the general case where ∂^*k*−1^*f*(*tP*_*k*_) takes the form of a power series, it may be approximated by ignoring powers (*t P*_*k*_)^*s*+1^ and higher, for some choice of *s*. This truncation corresponds to ignoring non-*k*-cycling walks of length greater than *s* in the original network. We emphasize, however, that in practice, for a specific choice of *f*, ∂^*k*−1^ *f*(*t P*_*k*_) is nothing but a matrix function [42] of *P*_*k*_; more efficient techniques than truncating a Taylor series are typically available to compute a matrix function, or its action on a vector. As they depend on the specific function, a full discussion is beyond the scope of this paper, and we refer the reader to the monograph [42] and the references therein.

Note that with this projection approach it is also possible to compute non-*k*-cycling *f*-total communicabilities as follows: (i) Compute *t*^*k*−1^∂^*k*−1^*f*(*tP*_*k*_)**1**; (ii) Project from the left by $L_{k-1}^{T}$; (iii) Add $t^{k-2}c_{k-2}p_{k-2;k}(A)1+\cdots +t^{2}c_{2}p_{2}(A)1+tc_{1}A1$.

Let us briefly comment on the last step of the approach described above. First, we note that there is no need to add the term *c*_0_**1**, as this addition would just produce a different representative of the same equivalence class under the relation in (5.1). The terms *tc*_1_*A***1** and *t*^2^*c*_2_*p*_2_(*A*)**1** = *t*^2^*c*_2_(*A*^2^ − *D*)**1** can be easily built from the data. As for the remaining terms *t*^ℓ^*c*_ℓ_*p*_ℓ;*k*_(*A*)**1** = *t*^ℓ^*c*_ℓ_*p*_ℓ;ℓ_(*A*)**1** for ℓ = 3, …, *k* − 2, these can be computed during the process of building the matrix *P*_*k*_. Indeed, it follows from theorem 5.5 and (5.2) that $p_{\ell ;\ell }(A)1=L_{\ell -1}^{T}P_{\ell }1$.

We observe that, although not necessarily tractable for large networks (see §5a), the procedure described above is mathematically well defined for any *k* ≥ 2.

We finally describe how to compute the non-backtracking and up to non-*k*-cycling *f*-subgraph centrality measure **x**_*k*_(*t*) in (5.1). It is readily seen from (5.4) that for all *i* = 1, 2, …, *n*:

$$\begin{array}{rl} {\left({\text{x}}_{k}(t)\right)}_{i} & =c_{0}+t^{k-1}{\text{e}}_{i}^{T}L_{k-1}^{T}[\partial^{k-1}f(tP_{k})]R_{k-1}{\text{e}}_{i}\\ & ∼{\text{e}}_{i}^{T}L_{k-1}^{T}[\partial^{k-1}f(tP_{k})]R_{k-1}{\text{e}}_{i}. \end{array}$$

As observed in theorem 4.3, the behaviour of the *f*-subgraph communicability is very different from that of the *f*-total communicability, in the sense that the former lacks ‘memory’; indeed, as mentioned in remark 5.1, the vector **x**_*k*_(*t*) only considers closed walks in assigning importance to the nodes in the network, and completely ignores closed walks whose length is less than *k* since they have already been removed at previous steps.

### (a) Remarks on complexity

This method can go on indefinitely, until we have removed cycles of length *n* (which is the length of the longest possible cycle). At that point, we have a method to count paths that can be used to define a path centrality.

Counting paths is #P-complete [47]. Therefore, if we had an algorithm for computing the *p*_*n*;*k*_(*A*) matrices in polynomial (in *n*) complexity even for *k* = *n*, this would imply that P = NP. Unfortunately for the authors, we do not have such an algorithm.

Observe that the size of the matrix *P*_*k*_ is equal to the number of *k*-plets of nodes in the input graph such that there is a path of length *k* − 1 through them. The worst case scenario is given by the complete graph with *n* nodes, for which there are *O*(*n*^*k*^) such *k*-plets. Therefore, even if all the subsequent steps are implemented in a complexity which is linear in the size, for *k* = *n* the method would yield an exponential complexity algorithm.

It should be noted, though, that it is entirely conceivable that for real-life networks, which are typically extremely sparse, this worst-case growth might not be relevant. This issue is followed up in §6.

### (b) Convergence

In this subsection we study the radius of convergence of the power series (5.4). This analysis will be used in §5, where we study universality properties of the centrality measures defined via the generating functions.

We begin by showing that the node space and generalized edge space series behave similarly.

**Lemma 5.7.**

*For all k, the series* $\Psi_{k}(t)=\sum_{r=0}^{\infty }c_{r}t^{r}P_{k}^{r}$ *and Φ*_*k*_(*t*) *in* (*5.3*) *have the same radius of convergence*.

**Proof.**

Denote by $\rho_{\Psi }$ and $\rho_{\Phi }$ the radii of convergence of Ψ_*k*_(*t*) and *Φ*_*k*_(*t*), respectively. Let $t<\rho_{\Psi }$. Therefore, by theorem 5.5

$$\Phi_{k}(t)={\tilde{\Phi }}_{k}(t)+t^{k-1}L_{k-1}^{T}\Psi_{k}(t)R_{k-1},\;\;{\tilde{\Phi }}_{k}(t):=\sum_{r=0}^{k-2}c_{r}t^{r}p_{r;k}(A).$$

Hence, the (*i*, *j*) entry of the sum *Φ*_*k*_(*t*) is equal to a finite sum plus an infinite sum. The latter is a linear combination of the entries of the absolutely convergent sum Ψ_*k*_(*t*). It follows that *Φ*_*k*_(*t*) converges, and hence, $\rho_{\Phi }\geq \rho_{\Psi }$.

Suppose now $t>\rho_{\Psi }$ and let $\left(r_{0},s_{0}\right)$ be such that ${\left(\Psi_{k}(t)\right)}_{r_{0}s_{0}}$ diverges. Moreover let (*i*_0_, *j*_0_) be such that ${\left(L_{k-1}\right)}_{r_{0}i_{0}}={\left(R_{k-1}\right)}_{s_{0}j_{0}}=1$; note that (*i*_0_, *j*_0_) is uniquely determined because $L_{k-1}$ and $R_{k-1}$ in (5.2) have precisely one nonzero element in each row. Observe that

$${\left(\frac{\Phi_{k}(t)-{\tilde{\Phi }}_{k}(t)}{t^{k-1}}\right)}_{i_{0}j_{0}}=\sum_{r,s}{\left(L_{k-1}\right)}_{ri_{0}}{\left(\Psi_{k}(t)\right)}_{rs}{\left(R_{k-1}\right)}_{sj_{0}}\geq {\left(\Psi_{k}(t)\right)}_{r_{0}s_{0}}$$

and *Φ*_*k*_(*t*) diverges. Hence, $\rho_{\Phi }\leq \rho_{\Psi }$ and we conclude that $\rho_{\Psi }=\rho_{\Phi }$. ▪

The next theorem characterizes the radius of convergence of (5.4) (via lemma 5.7) in terms of the number of cycles of length greater than *k*.

**Theorem 5.8.**

*For k* ≥ 2 *the spectral radius of the non-k-cycling matrix P*_*k*_ *of a simple and connected graph* G *satisfies the following properties*:

(i) *ρ*(*P*_*k*_) ≤ *ρ*(*P*_*k*−1_).
(ii) *If in* G *there are no cycles of length k then* *ρ*(*P*_*k*_) = *ρ*(*P*_*k*−1_).
(iii) *If in* G *there are no cycles of length greater than k then ρ*(*P*_*k*_) = 0.
(iv) *If in* G *there is precisely one cycle of length greater than k then ρ*(*P*_*k*_) = 1.
(v) *If in* G *there are at least two cycles of length greater than k then ρ*(*P*_*k*_) > 1.

Before proving this result, let us point out that any undirected cycle of length *k* in the original graph can be regarded as two directed cycles: one where the nodes are visited clockwise and one where the nodes are visited counterclockwise. It is readily seen that each of these two walks will also appear in the graphs associated to the matrices *P*_ℓ_ for all ℓ < *k*.

**Proof.**

(i) Elementwise it holds *p*_*r*;*k*_(*A*) ≤ *p*_*r*;*k*−1_(*A*) for all *r*, *k* and thus the statement follows from lemma 5.7.
(ii) This is a corollary of Flanders Theorem [37]. Since the graph contains no cycles of length *k*, then Δ_*k*_ = 0 and thus the matrices $P_{k-1}=L_{k-1}^{T}R_{k-1}$ and $P_{k}=W_{k}=R_{k-1}L_{k-1}^{T}$ have the same spectrum, up to the multiplicity of 0.
(iii) If there are no cycles of length greater than *k*, then the maximal length of non-*k*-cycling walks is finite. It follows that *Φ*_*k*_(*t*) in lemma 5.7 has only a finite number of nonzero addends, and hence it converges for all *t*. Thus, Ψ_*k*_(*t*) also converges for all *t* (and for all allowed choices of *f*) implying *ρ*(*P*_*k*_) = 0.
(iv) By the Gelfand formula, for any matrix norm ||⋅||,
  $$\rho (P_{k})=\lim_{r\to \infty }||P_{k}^{r}|{|}^{1/r};$$
  e.g. [48, corollary 5.6.14]. Note first that $\max_{i,j}|{\left(P_{k}^{r}\right)}_{ij}|\geq 1$, as there are two cycles in the graph of *P*_*k*_ and hence there exist walks of arbitrary length. We claim that, for *r* large enough, there exists a constant *c* ≥ 1, independent of *r*, such that $\max_{i,j}|{\left(P_{k}^{r}\right)}_{ij}|\leq c$. Since the latter is a matrix norm (not depending on *r*) of $P_{k}^{r}$, it follows that *ρ*(*P*_*k*_) = 1.
  It remains to prove the claim. Take *r* > *n* − *k*, where *n* is the number of nodes in the original graph. Then, any walk counted in $P_{k}^{r}$ contains at least one cycle. Only two cycles of length >*k* exist in the graph associated with *P*_*k*_, one corresponding to the cycle in the original graph being visited clockwise, and one corresponding to it being visited counterclockwise; clearly it is not possible for a walk to go from one to the other, since this would imply the existence of either (1) another cycle, longer than the one of length >*k* existing in the original graph, or (2) a cycle of length ≤*k* in the graph associated with *P*_*k*_. Case (1) leads to a contradiction, while (2) cannot happen because those walks have been removed at previous steps. This means that the walks we are considering must contain a number of consecutive circuits round one of the two cycles. Fix now i and j, two paths of length *k* − 1, and consider ${\left(P_{k}^{r}\right)}_{ij}$. This quantity is bounded above by a number $c_{ij}$ that can be constructed as the number of ways to enter one of the two cycles in the graph associated to *P*_*k*_ from i, times the number of ways to go from such cycle to j. These two numbers are finite, and do not depend on *r* but only on i and j. Taking $c=\max_{i,j}c_{ij}$ completes the argument.
(v) We first consider the case where two of the cycles of length greater than *k* in the original graph share at least one vertex, and denote their lengths by ℓ_1_ ≥ ℓ_2_ > *k*. Fix some integer *κ* ≥ ℓ_1_ + ℓ_2_ and let *s* be the integer satisfying
  $$s(\ell_{1}+\ell_{2})\leq \kappa <(s+1)(\ell_{1}+\ell_{2}).$$
  We will show that there is at least one entry of $P_{k}^{\kappa }$ that is bounded below by 2^*s*^, so that then
  $$\rho (P_{k})\geq \lim_{\kappa \to \infty }{\left(2^{s}\right)}^{1/\kappa }=2^{1/(\ell_{1}+\ell_{2})}>1$$
  and hence the conclusion in this case. Let us thus consider two non-(*k* − 1)-cycling walks in the original graph that belong to the first cycle. It is easily seen that there are at least
  (2ss)=(2s)!(s!)2≥2s
  non-*k*-cycling walks of length *κ* starting from one of these two paths and ending at the other. Indeed, there are *at least* that many non-*k*-cycling walks that go precisely *s* times around the first cycle and *s* times around the second cycle. Hence, for *κ* large enough, at least one entry of $P_{k}^{\kappa }$ is bounded below by 2^*s*^. Hence the conclusion. Suppose now that no pair of cycles share a vertex, then take any two cycles of length ℓ_1_ ≥ ℓ_2_ > *k*. These cycles are connected by (at least) one walk, whose length we denote by *d*. Fix now some *κ* ≥ 2 (ℓ_1_ + ℓ_2_ + *d*) and let *s* be the unique integer such that
  $$2s(\ell_{1}+\ell_{2}+d)\leq \kappa <(2s+1)(\ell_{1}+\ell_{2}+d).$$
  Let us count non-*k*-cycling walks that (1) start within the first cycle, (2) go precisely 2*s* times around the first cycles, 2*s* times around the second cycle, and *s* times back and forth on the bridge, and (3) end somewhere in the first cycle. There are at least
  22s−1(2s+1s+1)(2s−1s)≥22s+1
  such walks, and this gives a lower bound for at least one element of $P_{k}^{\kappa }$. It follows that
  $$\rho (P_{k})\geq \lim_{\kappa \to \infty }{\left(2^{2s+1}\right)}^{1/\kappa }=2^{1/(\ell_{1}+\ell_{2}+d)}>1.$$

 ▪

After some further analysis, we will go on to prove theorem 5.14, which shows that the converse of item (ii) in theorem 5.8 also holds. As the case *k* = 2 (note that ‘cycles of length exactly two’ are reciprocal edges, which are always present in a non-empty undirected graph) admits an easier proof, we treat it separately here.

**Proposition 5.9.**

*For any non-empty* (*i.e. there is at least one edge*) *simple graph with adjacency matrix A and Hashimoto matrix B, it holds that ρ*(*B*) < *ρ*(*A*).

**Proof.**

If the graph is a forest then *ρ*(*B*) = 0 < *ρ*(*A*). Assume that the graph is not a forest. Then, in view of the results in [24], we may assume that the graph is connected, and *ρ*(*B*) is equal to the largest finite eigenvalue of the matrix polynomial *I t*^2^ − *A t* + *D* − *I*. Moreover, by [24, theorems 4.7 and 6.1], the latter is invariant by iteratively removing all the leaves from the graph. On the other hand, the spectral radius of *A* can decrease by removing the leaves, but it cannot increase: hence, there is no loss of generality in assuming the graph has no leaves. We now argue similarly to [24, proof of theorem 4.8] and observe that

$$\rho {\left(B\right)}^{2}-\alpha \rho (B)+\beta =0,\;\alpha ={\text{v}}^{T}A\text{v},\;\beta ={\text{v}}^{T}D\text{v}-1,$$

where **v** ≥ 0 is such that **v**^*T*^**v** = 1 and (*Iρ*(*B*)^2^ − *Aρ*(*B*) + *D* − *I*)**v** = **0**. (Moreover, it is a consequence of the analysis in [24, section 9] that *ρ*(*B*) is the largest of the two roots of the above quadratic equation.) Note that *β* ≥ 1 [24, proof of theorem 4.8] and 0 ≤ *α* ≤ *ρ*(*A*). It follows that

$$2\rho (B)=\alpha +\sqrt{\alpha^{2}-4\beta }<2\rho (A).$$

 ▪

It is an immediate consequence of theorem 5.8 that for different values of *k* the ranges of the parameters *t* for which the generalized Katz centralities based on non-*k*-cycling walks, obtained via the procedure described in §5, consist of a sequence of nested intervals of the form

$$(0,\rho {\left(P_{1}\right)}^{-1})\subseteq (0,\rho {\left(P_{2}\right)}^{-1})\subseteq (0,\rho {\left(P_{3}\right)}^{-1})\subseteq \cdots \subseteq (0,\rho {\left(P_{k}\right)}^{-1})\subseteq \cdots .$$

Moreover, these intervals are strictly included in (0, 1) for all the values of *k* for which the (connected) graph contains at least two cycles of length greater than *k*; they are equal to (0, 1) for the values of *k* for which there is precisely one such cycle; and they are equal to (0, ∞) for all values of *k* such that there are no such cycles.

### (c) Generalized pruning

In this subsection, we show how the spectrum of *P*_*k*_ is invariant under certain pruning operations. These results are of direct interest, since they quantify the range of allowable values for the parameter *t*. They will also be used in the next subsection, where we study limiting behaviour.

Let G=(V,E) be a simple and connected graph, and let *k* ≥ 3 be a fixed path length. For the goals of this subsection, we partition the set of nodes for such fixed *k* into two subsets: V=B∪C. Here B is the minimal subset of nodes such that (1) given a fixed cycle-length *k* ≥ 3, all the cycles of length >*k* only visit nodes belonging to B and (2) each connected component in the subgraph spanned by C (if any) is connected to just one node in B (multiple connections to the same node in B are however allowed). We omit the trivial proof that, given *k*, such a partition exists and is unique, although in some cases one may have B=∅ or C=∅.

Let us point out that paths that originate in B and end in C cannot be prolonged *without introducing cycles of length ≤*k** to return to B, as this would imply the existence of a cycle of length >*k* outside B that we can use to cycle back.

Below, we will for simplicity use the verb ‘prolong’ to mean ‘prolong without introducing cycles of length ≤*k*’, as above. Consider now the following labelling of the open paths of length (*k* − 1) in G, i.e., of the row and column indices of the non-*k*-cycling matrix *P*_*k*_:

(i) paths in the original graph that start and end in B (and thus never leave B) and that can be prolonged into arbitrarily long walks;
(ii) paths in the original graph that start and end in B (and thus never leave B) and that cannot be prolonged into arbitrarily long walks;
(iii) all those paths that do not entirely take place within B and cannot be prolonged into arbitrarily long walks, thus cannot ‘return to B’; and finally
(iv) all the other paths: these do not entirely take place within B but can be prolonged into arbitrarily long walks, and hence will return to B in the limit.

With the described labelling, the matrix *P*_*k*_ can be written as a 4 × 4 block matrix; more specifically

5.5 $$P_{k}=\left[\begin{array}{cccc} Q_{k}(B) & ⋆ & ⋆ & 0\\ 0 & N_{1} & ⋆ & 0\\ 0 & 0 & N_{2} & 0\\ ⋆ & ⋆ & ⋆ & N_{3} \end{array}\right],$$

where *N*_*i*_ for *i* = 1, 2, 3 are nilpotent matrices of appropriate size. Indeed, the entries of the (1, 1) block, i.e. the entries of the matrix $Q_{k}(B)$ in (5.5), are non-zeros if and only if there are two walks within the subgraph spanned by the nodes in B that can be concatenated and indefinitely prolonged. The (2, 2) and (3, 3) blocks correspond to paths that are not indefinitely prolongable. Since it is possible to concatenate two such paths, the matrices in these blocks are not the zero matrix, in general. However, given the type of walks we are considering, powers of these matrices are going to be zero for large enough powers, since the walks are not indefinitely prolongable. Thus the matrices in blocks (2, 2) and (3, 3), denoted by *N*_1_ and *N*_2_, are nilpotent. A similar reasoning applies to the matrix *N*_3_ in the (4, 4) block. Indeed, there are only that many walks that do not entirely take place within B and that are prolongable to return to this set. For large enough values of *r*, all walks will have returned to B and thus $N_{3}^{r}=0$, and the matrix is nilpotent.

We now consider the off-diagonal blocks. The (2, 1) block is the zero matrix, since its entries record whether it is possible to concatenate walks that cannot be indefinitely prolonged with walks that can be indefinitely prolonged. The (1, 4) and (2, 4) blocks cannot have non-zero entries, as they would correspond to paths that take place in B entirely and are prolonged via paths that are not entirely on B but can return to this set. However this would imply the existence of a cycle outside B.

Blocks (3, 1), (3, 2) and (3, 4) correspond to walks that are not indefinitely prolongable and therefore cannot be connected, in the graph corresponding to *P*_*k*_, to any of the paths that are either taking place entirely within B or that can be prolonged to return to it.

**Remark 5.10.**

The non-*k*-cycling matrix associated with the subgraph of G spanned by B, which we denote by $P_{k}(B)$, is then

5.6 $$P_{k}(B)=\left[\begin{array}{cc} Q_{k}(B) & ⋆\\ 0 & N_{1} \end{array}\right].$$

The following theorem is an immediate consequence of the structure of the matrix *P*_*k*_.

**Theorem 5.11.**

*Let* G=(V,E) *be a simple connected graph and let k be a given cycle length. Let* V=B∪C *be partitioned as described at the beginning of this section and suppose that the edges are labelled as described above. Then, the spectrum of P*_*k*_, *the non-k-cycling matrix corresponding to* G, *coincides with that of* $Q_{k}(B)$ *in* (*5.6*) *up to the multiplicity of 0*.

Theorem 5.11 shows that, similarly to the NBT case *k* = 2 considered in [19,24], for *k* ≥ 3 the network dimension may be lowered by pruning in order to reduce the computational cost of finding the spectral radius of *P*_*k*_. The reciprocal of this spectral radius is a strict upper bound for the range of suitable *t* values in the non-*k*-cycling centrality measures.

**Remark 5.12.**

According to whether there are more than one, precisely one, or no cycles of length >*k*, the matrix $Q_{k}(B)$ above is, respectively, irreducible, permutation similar to a block diagonal matrix with two identical irreducible blocks, or empty. Therefore, the study of the spectral radius of *P*_*k*_ can be without loss of generality reduced to the case where the latter matrix is irreducible.

**Lemma 5.13.**

*Let P*_*k*_ *be partitioned as in* (*5.5*). *Then, for its right Perron eigenvector* **w** = *ρ*(*P*_*k*_)^−1^ *P*_*k*_**w**, *we have the coherent partition, with* **u**, **v** > 0,

$$\text{w}=\left[\begin{array}{c} \text{u}\\ 0\\ 0\\ \text{v} \end{array}\right].$$

*That is*, $w_{i}=0$ *if and only if* i *is a path of length* (*k* − 1) *that cannot be indefinitely prolonged*.

**Proof.**

We partition the nodes of the graph of the nonnegative matrix *P*_*k*_ into four categories, as described before theorem 5.11. Then, by remark 5.12, we can take $w_{i}>0$ if i is an indefinitely prolongable path that takes place entirely on B, i.e. if i belongs to category (i). From the fact that for large enough *r* (in particular, for *r* ≥ *R* where *R* is the maximum of nilpotency indices of *N*_1_, *N*_2_ and *N*_3_ in (5.5)), we have that

5.7 $$P_{k}^{r}=\left[\begin{array}{cccc} Q_{k}{\left(B\right)}^{r} & ⋆ & ⋆ & 0\\ 0 & 0 & 0 & 0\\ 0 & 0 & 0 & 0\\ ⋆ & ⋆ & ⋆ & 0 \end{array}\right],$$

it is clear that $w_{i}=0$ if i cannot be indefinitely prolonged (categories (ii) and (iii)). Finally, let i be a path of category (iv). Then, by remark 5.12, there exists a threshold *R* such that for *r* ≥ *R* and by the eigenequation defining **w**, we have

5.8 $$w_{i}=\rho {\left(P_{k}\right)}^{-r}\sum_{j}{\left(P_{k}^{r}\right)}_{ij}w_{j},$$

where the summation is taken over all paths j of length (*k* − 1) within G. By (5.7), if j is a path of type (iv), then ${\left(P_{k}^{r}\right)}_{ij}=0$. Moreover, if j is a path of either type (ii) or (iii), then $w_{j}=0$. Hence, the summation in (5.8) can be taken over all paths j of category (i) that can be connected to i in the graph associated with *P*_*k*_ via a path of length *r*. Suppose $w_{i}=0$: then, there is no such path j of category (i), for *no value* of *r* ≥ *R*. This contradicts the fact that i can be indefinitely prolonged, and hence, $w_{i}>0$. ▪

We are now in a position to prove the converse of item (ii) in theorem 5.8.

**Theorem 5.14.**

*If ρ*(*P*_*k*−1_) = *ρ*(*P*_*k*_) *then there are no cycles of length k*.

**Proof.**

We may assume without loss of generality that there are at least two cycles of length >*k*, otherwise the statement is a trivial corollary of items (iii), (iv) and (v) in theorem 5.8. Recall that by our construction there exist *L*_*k*−1_, *R*_*k*−1_, Δ_*k*_ such that $P_{k-1}=L_{k-1}^{T}R_{k-1}$, and $P_{k}=R_{k-1}L_{k-1}^{T}-\Delta_{k}$ and the absence of cycles of length *k* is tantamount to Δ_*k*_ = 0.

From the definition of *L*_*k*−1_ and Δ_*k*_ it follows that ${\left(L_{k-1}^{T}\Delta_{k}\right)}_{ij}=1$ if the (*k* − 2)-path i in G is part of a *k*-cycle and can be prolonged within this cycle to form the (*k* − 1)-path j, while ${\left(L_{k-1}^{T}\Delta_{k}\right)}_{ij}=0$ otherwise. Suppose that *ρ*(*P*_*k*_) = *ρ*(*P*_*k*−1_) = *ρ* > 1. Then for a left Perron eigenvector **a** of *P*_*k*−1_ and a right Perron eigenvector **w** of *P*_*k*_ the following equations hold:

$${\text{a}}^{T}(L_{k-1}^{T}R_{k-1})=\rho {\text{a}}^{T},\;\;(R_{k-1}L_{k-1}^{T})\text{w}=\Delta_{k}\text{w}+\rho \text{w}.$$

Combining the equations above we thus see that

5.9 $${\text{a}}^{T}L_{k-1}^{T}\Delta_{k}\text{w}=\sum_{i,j}a_{i}w_{j}=0,$$

where the sum is taken over all pairs (i,j) such that ${\left(L_{k-1}^{T}\Delta_{k}\right)}_{ij}\neq 0$, i.e. the (*k* − 2)-path i is part of a *k*-cycle and can be prolonged within such a cycle to make the (*k* − 1)-path j. By remark 5.12, we can assume that *P*_*k*−1_ is irreducible, and hence, **a** > 0. It is worth stressing that in this context we cannot simultaneously make the same assumption on *P*_*k*_: we only know **w** ≥ 0. We therefore conclude that either the summation in (5.9) is empty, and hence there is no cycle of length precisely *k*, i.e., Δ_*k*_ = 0, or $w_{j}=0$ for *all* open paths of length *k* − 1 that are part of a *k*-cycle. We claim that the latter is impossible: if there is a cycle of length *k* in the original graph then there is at least one such open path, labelled $j_{0}$ in the graph of *P*_*k*_, such that $w_{j_{0}}\neq 0$. This claim proves the statement.

To prove the claim, let us partition the nodes V=B∪C for fixed *k* as described at the beginning of this subsection. We further partition the (*k* − 1)-paths in G, i.e. the nodes of the graph of *P*_*k*_, into the same four categories described before theorem 5.11. Suppose that there exists a cycle of length *k* in G whose nodes all belong to B. Then there exists a (*k* − 1)-path $j_{0}$ that is indefinitely prolongable and belongs to this cycle. It thus belong to category (i) and hence $w_{j_{0}}>0$ by lemma 5.13. Suppose now that the cycle of length *k* contains at least one node in C. From the definition of B and C it follows that there is at most one node in the cycle that belongs to B, as otherwise we would have a connected component in the graph spanned by C that is connected to at least two nodes in B. Hence, there is at least one open path $j_{0}$ of length (*k* − 1) that belongs to such a cycle and does not entirely take place within B, but can be indefinitely prolonged, i.e. is of category (iv). By lemma 5.13 we again have $w_{j_{0}}>0$. This proves the claim and hence the theorem. ▪

### (d) Non-*k*-cycling centralities and universality classes

We now extend the results in theorem 4.3 to the case of non-cycling walks. In summary, we find that the limiting behaviour for subgraph centrality measures does not depend on the underlying scalar function *f*(*x*). However this is not true for the case of total communicability; here, in the generic case (iii) in theorem 5.15, this quantity is seen to depend on the coefficients *c*_1_, *c*_2_, …, *c*_*k*_. An important corollary of this result is that, unlike in the NBT case *k* = 2 studied in [19,24], there can be no universal eigenvector-based non-cycling centrality measure arising as the limit of the walk-counting version.

**Theorem 5.15.**

*Let c*_*r*_ > 0 *for all r, and assume that the underlying graph is simple and connected. Consider the centrality measures* **x**_*k*_(*t*) *and* **y**_*k*_(*t*) *in* (*5.1*) *for k* > 2. *Suppose that the power series converge with radii of convergence* ${\bar{t}}_{k}.$ *Then, in the limit t* → 0 *we have*

$${\text{x}}_{k}(t\to 0)∼\left\{ \begin{array}{ll} 1 & \text{if there are no cycles of length}>k\\ {\text{d}}^{\left(\ell \right)} & \text{otherwise} \end{array}\right.\;\text{and}\;{\text{y}}_{k}(t\to 0)∼\text{d},$$

*where ℓ* > *k is the length of the shortest cycle that can be traversed, if any*, **d**^(ℓ)^ *is the vector whose ith entry is the number of cycles of length ℓ centred at node i, and* **d** *is the vector of degrees. Moreover*,

(i) *if the graph does not contain any cycle of length* >*k, then* ${\bar{t}}_{k}=\infty$ *and* ${\text{x}}_{k}(t\to {\bar{t}}_{k})∼1$ *and* **y**_*k*_(*t* → ∞) ∼ *p*_*h*+*k*−1;*k*_(*A*)**1**, *where h is the length of the longest path in P*_*k*_.
(ii) *if the graph contains exactly one cycle of length* >*k*, *then* ${\text{x}}_{k}(t\to {\bar{t}}_{k})$ *only depends on the distance of each node from the cycle of length* >*k, while* ${\text{y}}_{k}(t\to {\bar{t}}_{k})∼1$.
(iii) *if the graph contains at least two cycles of length* >*k, then* ${\text{x}}_{k}(t\to {\bar{t}}_{k})$ *and* ${\text{y}}_{k}(t\to {\bar{t}}_{k})$ *exist and are unique. The limit vector* ${\text{x}}_{k}(t\to {\bar{t}}_{k})$ *depends on k, but not on the choice of the coefficients c*_*r*_. *Similarly, the shifted limit vector* ${\text{y}}_{k}(t\to {\bar{t}}_{k})-(c_{0}1+c_{1}\text{d}+\cdots +c_{k-1}p_{k-1;k}(A)1)$ *depends on k, but not on the choice of the coefficients c*_*r*_.

**Proof.**

The limit *t* → 0 can be analysed straightforwardly, as was the case in theorem 4.3. For the limit for $t\to {\bar{t}}_{k}$, there are three cases:

(i) If the graph does not contain any cycle of length >*k*, then the graph associated with *P*_*k*_ is a cycle-less digraph, and hence ${\bar{t}}_{k}=\infty$ and **x**_*k*_(*t* → ∞) ∼ **1**. Moreover, if we let *h* be the length of the longest directed path in the graph associated with *P*_*k*_, then **y**(*t* → ∞) ∼ *p*_*h*+*k*−1;*k*_(*A*)**1**; see theorem 5.5.
(ii) If the graph contains exactly one cycle of length ℓ > *k*, the matrix *P*_*k*_ has 1 as its eigenvalue, with algebraic and geometric multiplicity two. Indeed, using the same partition of nodes described in §5c and the labelling of paths of length (*k* − 1) described in theorem 5.11, it follows that $\Lambda (P_{k})=\Lambda (P_{k}(B))\cup \{0\}$. It remains to describe $P_{k}(B)$. By the remarks in §5c we can focus on studying $Q_{k}(B)$. It is clear that anything that touches any shortcut is not indefinitely prolongable. Hence, up to permutation similarity, $Q_{k}(B)=C⊕C^{T}$ where $C\in R^{\ell \times \ell }$ is the circulant adjacency matrix of a directed cycle. It immediately follows that 1 is an eigenvalue of *P*_*k*_ with both algebraic and geometric multiplicity 2.
  The conclusion then follows using a similar reasoning to that of theorem 4.3 (ii).
(iii) Finally, suppose that the graph contains at least two cycles of length >*k*. The matrix *P*_*k*_ is then permutation similar to (5.5), where the matrix $P_{k}(B)\neq 0$ in (5.6) is now nonnegative and irreducible. Therefore, it follows from the Perron–Frobenius theorem that the spectral radius of $P_{k}(B)$, and hence of *P*_*k*_, is a simple positive eigenvalue. The conclusion then follows from a similar reasoning to that of theorem 4.3 (i). ▪

## 6. Tests on real data

In this section we record the dimension (number of rows/columns), the number of nonzero elements, and density of the square matrices *P*_1_ = *A*, *P*_2_ = *B*, *P*_3_, *P*_4_ for some example networks. Our aim is to get a feel for the growth of these quantities as a function of the initial network size, *n*. We first consider samples from widely used random graph models, where testing over a range of *n* is straightforward. We then take real, fixed, network data and work on increasingly large subgraphs.

We begin by pointing out some relevant analytical results. For any undirected graph G with *n*_1_ : = *n* nodes and *m*_1_ : = *m* (directed) edges, the number of nonzeros in $P_{2}\in R^{n_{2}\times n_{2}}$, where *n*_2_ = *m*_1_, corresponds to twice the number of undirected open paths of length two in G; so, (e.g. [49]) *m*_2_ = **d**^*T*^(**d** − **1**), where we recall that **d** = (*d*_*i*_) is the vector of degrees. The non-backtracking, non-triangulating matrix $P_{3}\in R^{n_{3}\times n_{3}}$, where *n*_3_ = *m*_2_, has a number of nonzeros that corresponds to twice the number of undirected open paths of length three in G, so

$$n_{3}=\sum_{(i,j)\in E}(d_{i}-1)(d_{j}-1)-2\cdot 3\left(\frac{1}{6}\text{tr}(A^{3})\right)={\left(\text{d}-1\right)}^{T}A(\text{d}-1)-\text{tr}(A^{3}),$$

where the summation was taken over the *m*_1_ directed edges in G. We define the density *δ*_*k*_ of the matrix *P*_*k*_ for *k* = 1, 2, 3, … as *δ*_*k*_ = *m*_*k*_/(*n*_*k*_(*n*_*k*_ − 1)).

**Figure 2.**
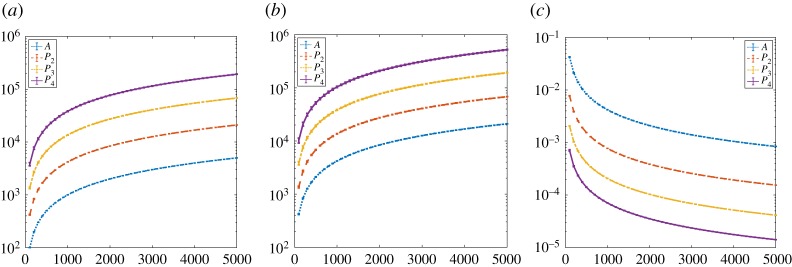
Evolution of average dimension, number of nonzeros and density of the non-*k*-cycling matrices *P*_*k*_ for *k* = 1, 2, 3, 4 corresponding to small-world networks of increasing size *n* = 100, 200, …, 5000. (*a*) Dimension, (*b*) nonzeros, and (*c*) density. (Online version in colour.)

**Figure 3.**
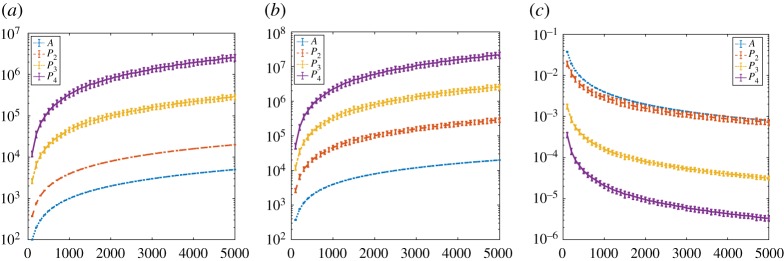
Evolution of average dimension, number of nonzeros and density of the non-*k*-cycling matrices *P*_*k*_ for *k* = 1, 2, 3, 4 corresponding to preferential attachment networks of increasing size *n* = 100, 200, …, 5000. (*a*) Dimension, (*b*) nonzeros, and (*c*) density. (Online version in colour.)

In figure 2 we display on a semi-logarithmic scale (a) the evolution of the dimension *n*_*k*_ of the matrices *P*_*k*_ for *k* = 1, 2, 3, 4, (b) the evolution of the number of nonzeros *m*_*k*_, and (c) that of their densities *δ*_*k*_ for networks of increasing size built using the smallw function from the CONTEST toolbox for Matlab [50], with default parameters. The function smallw(n) returns the adjacency matrix of an independent sample from a class of small world networks [51] with *n* nodes. In our tests, we selected *n* = 100, 200, …, 4900, 5000. For each of these we have computed the dimensions of the matrices *P*_*k*_ for *k* = 1, 2, 3, 4, the number of nonzeros, and their densities; we ran this test 100 times and averaged the results. Error bars are also shown in the plots to indicate the standard errors. The same test was run for networks of increasing size built using a preferential attachment type of model [52]: pref(n), for the same values of *n*. Results are displayed in figure 3.

For these two widely used models, it can be seen that although the dimension of the matrices *P*_2_, *P*_3_ and *P*_4_ increases considerably with the size of the original network, they remain very sparse, thus allowing for fast computations.

**Figure 4.**
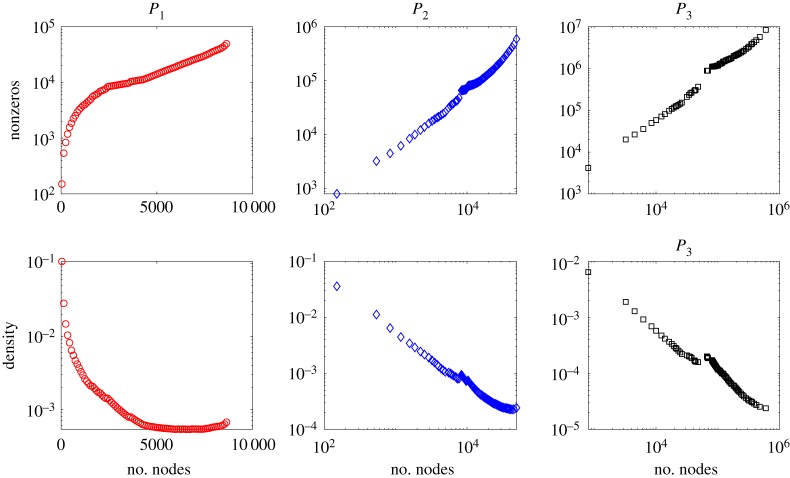
Evolution of number of nonzeros and of the density of the matrices *P*_1_ = *A*, *P*_2_, and *P*_3_ associated with subgraphs of the largest connected component of the network ca-HepTh. (Online version in colour.)

**Figure 5.**
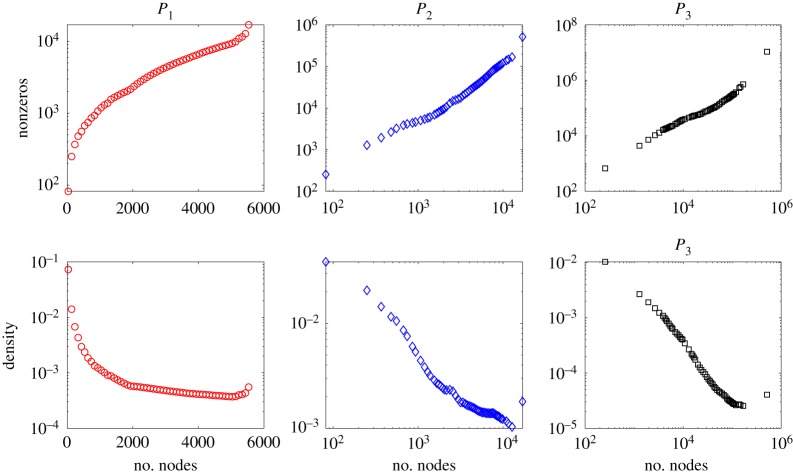
Evolution of number of nonzeros and of the density of the matrices *P*_1_ = *A*, *P*_2_, and *P*_3_ associated with subgraphs of the largest connected component of the network erdos02. (Online version in colour.)

For our tests on real world networks, we raise the dimension by constructing increasingly large, well-connected subsets of a fixed network. To do this, we first compute the Fiedler vector of the largest connected component. Since the Fiedler vector is an eigenvector of the graph Laplacian, it is defined up to scalar, nonzero multiples. We retained the sign returned when the eigenvector was computed using the MATLAB built-in function eigs and we selected the nmod  100^1^ nodes corresponding to the largest positive entries in the Fiedler vector. We iterated this process by adding, at each new step, 100 more nodes to the subgraph using the ordering of the nodes induced by the Fiedler vector, until we reached the size of the largest connected component of the original graph. Since close components in the Fiedler are good candidates for members of the same cluster [53], this process is designed to run through well connected neighbourhoods. The dimension and density of *P*_1_, *P*_2_ and *P*_3_ are displayed in figure 4 for the largest connected component of the collaboration network ca-HepTh (*n* = 8638) and in figure 5 for the largest connected component of the collaboration network Erdos02 (*n* = 5534). Both networks are available at [54]. On the *x*-axis we display the dimension of *P*_1_, *P*_2_ and *P*_3_ and on the *y*-axis we display the number of nonzeros (top plots) and the density (bottom plots). The results associated with *P*_1_ are plotted in a semi-logarithmic scale, while the results for *P*_2_ and *P*_3_ are displayed in log–log plots. Again, we observe that the non-*k*-cycling matrices are rather sparse for these real world networks.

## 7. Conclusion

Motivated by the wide application of non-backtracking walks, our aim here was a natural extension of this concept to the case of non-triangulating, non-squaring and, in general, the elimination of all cycles. From a practical perspective, we showed that recursively unfolding the Hashimoto matrix construction provides building blocks for the required generating functions and non-cycling walk centralities. We also developed a range of theoretical results that characterize the spectra of the associated matrices and the limiting behaviour of the centrality measures.

We hope that this new computational and analytical framework will initiate further study in areas where non-backtracking walks have proved attractive. In particular, for the network science setting of this work, we envisage progress in a number of directions, including : development of spectral results, such as the decay of *ρ*(*P*_*k*_) in part (i) of theorem 5.8 as *k* increases, for specific graph classes, and their consequences in terms of localization of centrality measures; fast linear algebra algorithms that can exploit the structure of the matrix-based subproblems, including the evaluation of general power series in *tP*_*k*_; large-scale tests of the new network science measures on real-life complex networks of current research interest in science and technology.

## Supplementary Material

Supplementary Summary

## Supplementary Material

matlab files updated

## Footnotes

1 We selected 100 nodes if nmod  100=0.

## Data accessibility

MATLAB codes associated with all computational experiments is made available as supplementary material. This article has no additional data.
